# Time‐Decoupled Electrolysis via a Rechargeable Metal‐Urea Battery for Waste Urea Treatment and Hydrogen Production

**DOI:** 10.1002/advs.202507657

**Published:** 2025-07-17

**Authors:** Dong Lv, Zhengrong Xu, Xingyu Guo, Deng Liu, Rui Liu

**Affiliations:** ^1^ Key Laboratory of Advanced Civil Engineering Materials of Ministry of Education School of Materials Science and Engineering Tongji University Shanghai 201804 China

**Keywords:** battery, electrocatalyst, hydrogen, temporal decoupling, urea

## Abstract

The resource utilization of urea wastewater is an important issue in the environmental field. In the present work, urea can be used as the resource of pure hydrogen production by a temporally decoupled rechargeable metal‐urea battery. During the cathodic charging process, urea is dissociated by the reaction of CO(NH_2_)_2_ + 6OH^−^→ N_2_ + 5H_2_O + CO_2_ + 6e^−^ to achieve the disposal of urea. Water is splitting into hydrogen (2H_2_O + 2e^−^→ H_2_ + 2OH^−^) during the cathodic discharging process, accompanied with the production of electrochemical energy. The key catalyst layers at the cathode employed bifunctional Ni/Mo_2_C electrocatalysts for both the urea oxidation reaction (UOR) and the hydrogen evolution reaction (HER). The home‐made Zn–Urea battery can accomplish continuous hydrogen production with a Faraday efficiency of 99% over 20 h, together with a maximum power density of 3.4 mW cm^−2^. The temporally decoupled rechargeable metal‐urea battery can convert waste urea into high‐value purified hydrogen with partial recovery of electrical energy, offering an impressive resource utilization route for wastewater treatment.

## Graphical abstract

A rechargeable metal‐urea battery is reported with a time‐decoupled splitting mechanism, separating urea oxidation during charging and hydrogen evolution during discharging, to eliminate kinetic mismatches and boost efficiency. The development of the temporally decoupled rechargeable metal‐urea battery for hydrogen production not only addresses urea waste disposal, but also contributes to renewable energy generation.

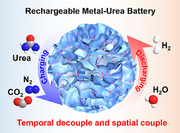

## 1 Introduction

The urea wastewater discharged from industrial and agricultural production as well as urban life is characterized by high concentrations, high ammonia nitrogen levels, and biological toxicity.^[^ ^1^, ^2^, ^3^ ^]^ The untreated urea wastewater would pose a serious threat to the ecological environment and human health, such as water eutrophication, deterioration of water quality and formation of ecological dead zones.^[^ ^4^, ^5^, ^6^ ^]^ Traditional techniques including physical adsorption, thermal hydrolysis, and the urease method have been applied to treat urea wastewater.^[^ ^7^, ^8^, ^9^ ^]^ Though existing some efficiencies, these processes may consume a great deal of energy, materials or enzyme, and are difficult to recover valuable economic products. Therefore, it is highly desired to develop an effective resource utilization method for urea wastewater treatment.

Hydrogen produced by electrochemically splitting water with renewable electric power is a promising energy storage and conversion carrier.^[^ ^10^, ^11^, ^12^, ^13^, ^14^, ^15^, ^16^ ^]^ The key anode oxygen evolution reaction (OER) during electrochemical water splitting exhibits significant kinetic barriers, resulting in an actual input voltage exceeding 1.5 V in the electrolytic cell.^[^ ^17^, ^18^, ^19^, ^20^, ^21^, ^22^ ^]^ The produced O_2_ at the anode not only degrades ion‐exchange membranes with the generated reactive oxidation species, but also poses safety risks owing to easy formation of explosive and flammable mixtures with hydrogen.^[^ ^23^, ^24^ ^]^ In contrast, urea oxidation reaction (UOR) emerges as an attractive alternative anodic reaction pathway, owing to its significantly lower oxidation potential of 0.37 V (vs RHE) than that of OER (1.23 V vs RHE).^[^ ^25^, ^26^, ^27^ ^]^ Additionally, UOR exclusively produces inert and environmentally benign nitrogen and carbon dioxide, also representing a promising approach for urea wastewater treatment.^[^ ^28^, ^29^, ^30^ ^]^ Recent advances in nanostructured electrocatalysts have demonstrated significant potential in enhancing the efficiency and stability of hydrogen production, UOR and other electrochemical processes. Noteworthy examples include heterostructures, nanoclusters, and transition metal‐based catalysts, which have exhibited exceptional catalytic activity and durability for electrocatalysis.^[^ ^31^, ^32^, ^33^, ^34^ ^]^

**Figure 1:**
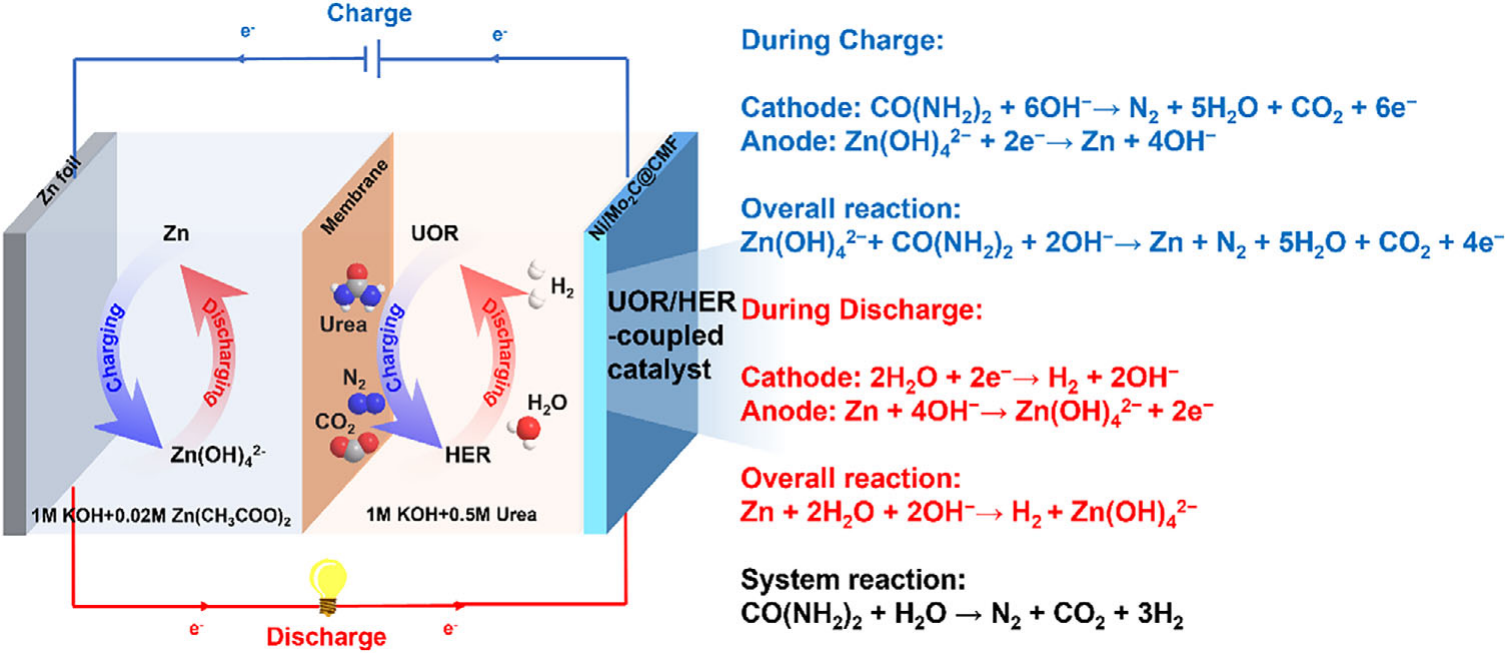
Schematic illustration of Zn–Urea battery with hydrogen generation and urea decomposition via decoupled UOR and HER at charge/discharge processes.

Direct electrolysis of urea solutions results in a mixture of hydrogen, nitrogen, and carbon dioxide, complicating the separation and purification of hydrogen and increasing the operational cost.^[^ ^35^, ^36^, ^37^, ^38^, ^39^, ^40^, ^41^, ^42^, ^43^, ^44^, ^45^ ^]^ An effective electrolysis strategy is expected to naturally separate hydrogen from urea electrolysis gases, which may facilitate the production of purified hydrogen while simultaneously decomposing urea. Recent advancements proposed rechargeable metal‐catalysis batteries, such as Zn–NH_3_ and Zn–hydrazine batteries, which enabled efficient hydrogen production without the need for gas separation or purification. These systems achieved hydrogen generation through time‐decoupled oxidation and hydrogen evolution reactions.^[^ ^37^, ^38^ ^]^ Herein, a rechargeable metal‐urea battery is developed for efficient urea‐to‐hydrogen conversion, featuring a time‐decoupled splitting process (**Figure** **1**). In this temporal decoupling system, urea oxidation to nitrogen and carbon dioxide occurs during charging the battery, while hydrogen evolution from water takes place during discharging the battery. It actually enables hydrogen production without the need for separation or purification by decoupling UOR and HER over time scale. The bifunctional Ni/Mo_2_C electrocatalyst layers are specially constructed to accelerate UOR/HER and match their electrocatalytic efficiency, together with adjusting the metal contrast electrodes. The originality of this work lies in the innovative design of a rechargeable metal‐urea battery that effectively decouples urea oxidation and hydrogen evolution reactions, enabling continuous hydrogen production without the need for gas separation or purification. Additionally, this approach offers an efficient method to convert urea into high‐purity hydrogen with a Faraday efficiency of 99% over 20 h, providing a sustainable solution for simultaneous waste treatment and clean energy generation.

## 2 Results and Discussion

### 2.1 Synthesis and Characterization of Bifunctional Catalyst

**Figure 2:**
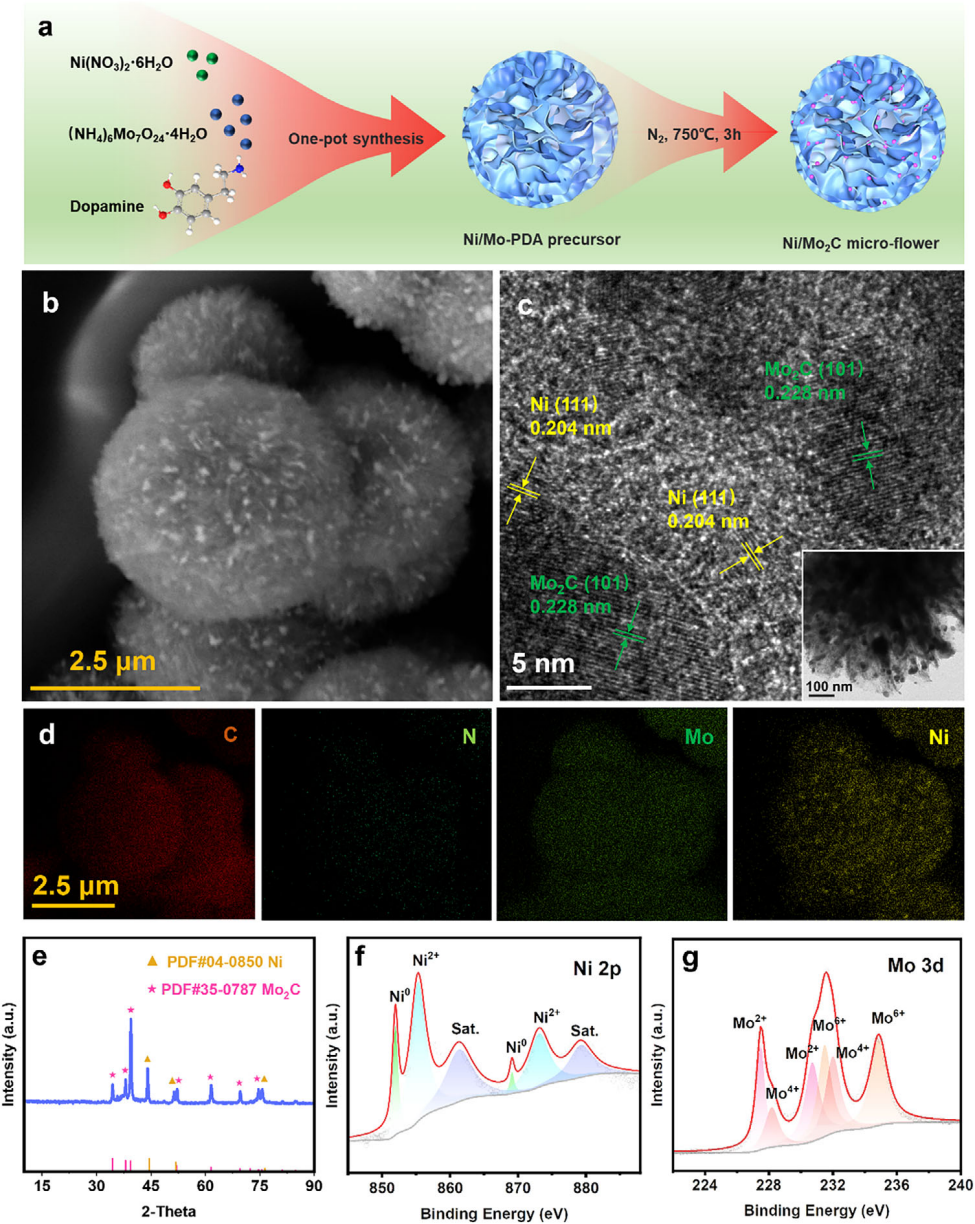
a) Schematic synthetic route for Ni/Mo_2_C@CMF. Characterization of Ni/Mo_2_C@CMF: b) SEM, c) HRTEM and TEM (inset), d) EDS mapping images, e) XRD pattern, XPS spectra of f) Ni 2p and g) Mo 3d.

A dual‐functional electrocatalyst for both UOR and HER is essential to construct Metal‐Urea batteries. Nickel and molybdenum transition metals were specially chosen because of the impressive catalytic activities for UOR over Ni and HER over Mo_2_C.^[^ ^43^, ^44^, ^45^ ^]^ In the typical synthesis of electrocatalyst, Ni/Mo‐polydopamine (PDA) precursors were produced via one‐pot polymerization, which were then annealed at 750 °C to obtain Ni/Mo_2_C@CMF (**Figure** **2**a). The formed Ni and Mo_2_C nanoparticles were evenly embedded on the ≈2 µm‐sized carbon micro‐flowers (Figures 2b; Figure S1a, Supporting Information). For comparison, polydopamine, Ni‐polydopamine, and Mo‐polydopamine precursors were carbonized, giving the corresponding carbon sphere (PDA‐CS), irregular Ni@C nanoparticles, and flower‐like Mo_2_C@CMF, respectively (Figure S1b–d, Supporting Information). C and N elements were uniformly distributed over PDA‐CS. In addition of C and N, uniform distributions of Ni and Mo were also observed over Ni@C and Mo_2_C@CMF, respectively. The contrast morphology showed that the inclusion of (NH_4_)_6_Mo_7_O_24_·4H_2_O could facilitate the formation of a flower‐like structure in both Mo_2_C@CMF and Ni/Mo_2_C@CMF.^[^ ^46^ ^]^ The microstructure of Ni/Mo_2_C@CMF was further characterized by transmission electron microscopy (TEM), clearly showing ≈10 nm nanoparticles dispersed on the carbon micro‐flowers (Figure 2c inset). A clearer observation for these nanoparticles by high‐resolution TEM (HRTEM) images indicated the well‐defined crystalline stripes of Ni (111) (0.204 nm) and Mo_2_C (101) (0.228 nm), generating abundant interfaces or grain boundary defects between them (Figure 2c). C, N, Mo, and Ni elements were uniformly distributed over Ni/Mo_2_C@CMF (Figure 2d).

The X‐ray diffraction (XRD) pattern of Ni/Mo_2_C@CMF (Figure 2e) revealed that the diffraction peaks at 44.5°, 51.8°and 76.3° were corresponded to (111), (200) and (220) planes of metallic nickel (JCPDS No. 04–0850), while peaks at 34.4°, 38.0°, 39.4°, 52.1°, 61.5°, 69.6°, and 74.6° were assigned to (100), (002), (101), (102), (110), (103) and (112) planes of Mo_2_C (JCPDS No. 35–0787). The XRD patterns (Figure S2, Supporting Information) of Ni@C and Mo_2_C@CMF exhibited the characteristic peaks corresponding to metallic nickel and Mo_2_C, respectively, whereas PDA‐CS displayed only the characteristic peaks of amorphous carbon. X‐ray photoelectron spectroscopy (XPS) survey spectra further confirmed the surface species consisting of C, N, O, Ni, and Mo in Ni/Mo_2_C@CMF (Figure S3a, Supporting Information). In the Ni 2p spectrum (Figure 2f), the distinct peaks at 852.0 eV and 870.1 eV were originated from Ni^0^, while the peak at 855.3 eV was assigned to Ni^2+^ derived from partly oxidation at the surface.^[^ ^43^ ^]^ The Mo 3d spectrum (Figure 2g) showed three different Mo species, including Mo^2+^, Mo^4+^, and Mo^6+^. The binding energies at 227.5 and 230.8 eV were owing to Mo^2+^, referring to Mo_2_C.^[^ ^37^ ^]^ The Mo^4+^ and Mo^6+^ species should originate from the MoO_2_ and MoO_3_ components, which could be ascribed to partial surface oxidation.^[^ ^43^ ^]^ Additionally, the C 1s XPS spectrum was deconvoluted into three peaks at 286.1, 284.3, and 283.6 eV, referring to C─O, C─C, and Mo─C, respectively^[^ ^37^ ^]^ (Figure S3b, Supporting Information). The N 1s XPS spectrum in Figure S3c (Supporting Information) was deconvoluted into three peaks at 397.9, 398.9, and 400.1 eV, referring to pyridinic, pyrrolic, and graphitic N, respectively. These results confirmed the formation of Ni/Mo_2_C nanoparticles embedded on flower‐like carbons with abundant defects and partially oxidized Ni and Mo species.

### 2.2 UOR Catalytic Activity

**Figure 3:**
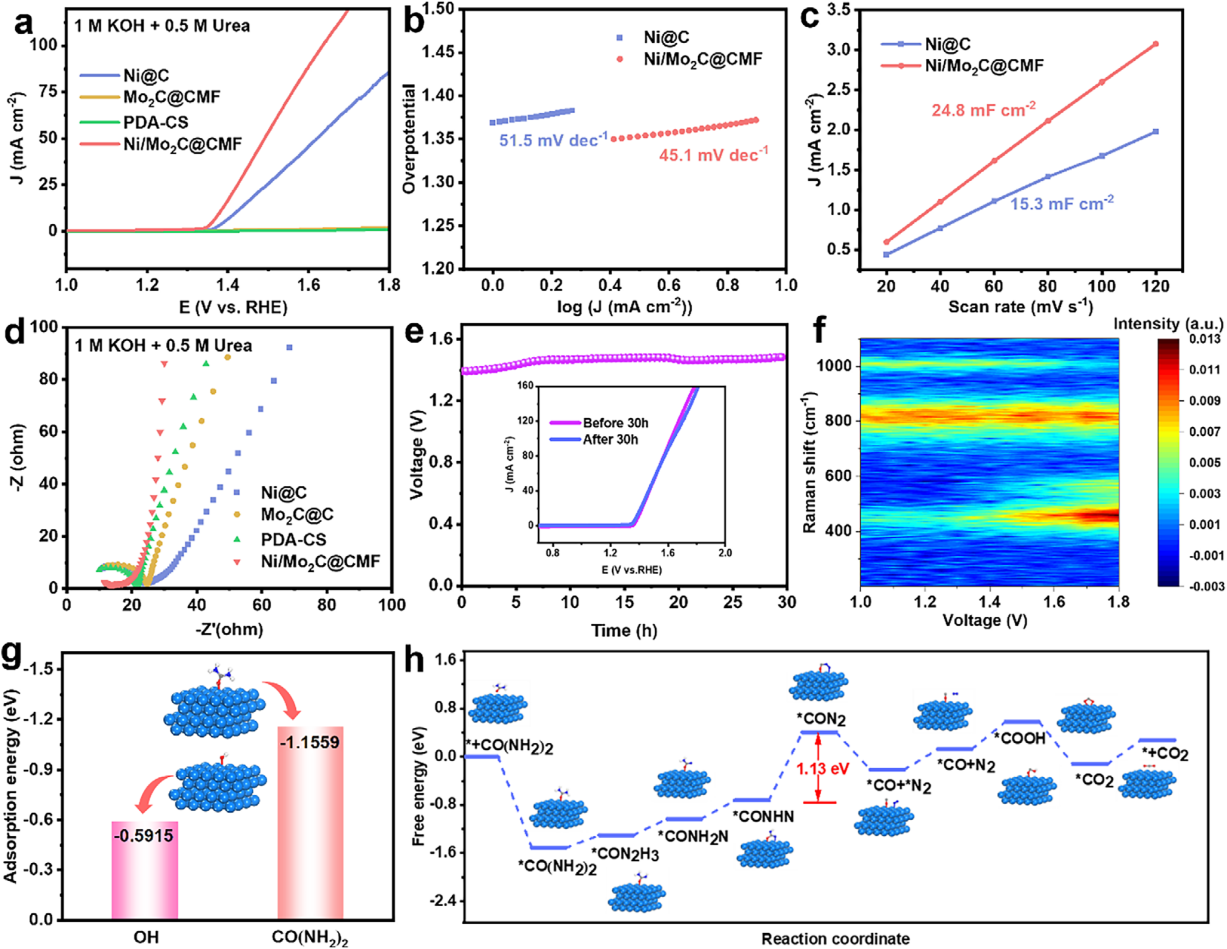
UOR catalytic performance: a) LSV curves of Ni@C, Mo_2_C@CMF, PDA‐CS and Ni/Mo_2_C@CMF, b) Tafel curves and c) C_dl_ of Ni@C and Ni/Mo_2_C@CMF, d) Nyquist plot of Ni@C, Mo_2_C@CMF, PDA‐CS and Ni/Mo_2_C@CMF, e) chronopotentiometry curve at the current density of 10 mA cm^−2^. (Inset: polarization curves before and after 30 h, f) in situ Raman spectra, g) adsorption energy of CO(NH_2_)_2_^*^ and OH^*^on Ni(111), h) calculated Gibbs free energy diagrams of UOR steps on Ni(111).

The electrocatalytic performances of Ni/Mo_2_C@CMF were first evaluated in 1.0 m KOH with 0.5 m urea solution and 1.0 m KOH, respectively. As depicted in Figure S4 (Supporting Information), the enhanced catalytic activity of Ni/Mo_2_C@CMF for UOR was highlighted by a substantial reduction in the applied voltage (194 mV) to reach the same current density of 10 mA cm^−2^, in contrast to OER performance in 1.0 m KOH. It proved that the urea oxidation reaction occurred preferentially in the presence of urea.^[^ ^47^ ^]^ Then, a series of electrochemical tests were conducted in a half‐cell system under identical electrolytic conditions (1 m KOH and 0.5 m urea) to evaluate its dual‐functional performance. The linear sweep voltammetry (LSV) curves of various materials are shown in **Figure** **3**a. Ni/Mo_2_C@CMF exhibited the most prominent UOR activity with a voltage value of 1.379 V at a current density of 10 mA cm^−2^. Notably, both PDA‐CS and Mo_2_C@CMF showed negligible UOR activity, thus confirming that Ni was served as the exclusive active site for UOR. Though active for UOR, the lower current density of Ni@C (1.419 V at 10 mA cm^−2^) further highlighted that the better performance of Ni/Mo_2_C@CMF may be attributed to the accessibility of active sites with a flowerlike structure. Consequently, Ni/Mo_2_C@CMF exhibited a remarkably low Tafel slope of 45.1 mV dec^−1^, indicating a rapid kinetic rate (Figure 3b). The electrochemical double‐layer capacitance (C_dl_) is directly proportional to the electrochemical surface area (ECSA), which is determined from the linear slope based on current density and scan rate. As shown in Figure 2c and Figure S5 (Supporting Information), Ni/Mo_2_C@CMF displayed a C_dl_ of 24.8 mF cm^−2^, corresponding to an ECSA of 620 cm^2^. ECSA‐normalized LSV of Ni/Mo_2_C@CMF revealed the highest ECSA‐normalized current density (Figure S6, Supporting Information). The EIS data for Ni/Mo_2_C@CMF, PDA‐CS, Mo_2_C@CMF, and Ni@C showed that Ni/Mo_2_C@CMF (Figure 3d) exhibited the lowest charge‐transfer resistance (R_ct_) among these catalysts, indicating rapid interfacial dynamics. Furthermore, this electrocatalyst also demonstrated excellent stability at a current density of 10 mA cm^−2^ with only a slight increase in voltage over 30 h (Figure 3e and inset). As shown in Figure S7a,b (Supporting Information), the XRD pattern and SEM image confirmed that Ni and Mo_2_C phase structure remained and Ni/Mo_2_C@CMF retained its micro‐flower morphology after the stability test.

Raman characterization of Ni/Mo_2_C@CMF without urea was first tested to investigate the origin of C─N bonds for comparison. As shown in Figure S8 (Supporting Information), the Raman spectrum without urea showed almost no peak at 1000 cm^−^¹ while the presence of urea brought a peak at 1000 cm^−^¹. This difference indicated that the peak near cm^−^¹ should originate from urea rather than C─N bonds formed during dopamine pyrolysis. To gain a deeper understanding of reactions over Ni/Mo_2_C@CMF, in situ Raman spectroscopy was performed within the potential range of 1.0 to 1.8 V (vs RHE). As shown in Figure 3f and Figure S9 (Supporting Information), the characteristic peak of C─N bond at 1000 cm^−1^ notably diminished with increasing voltage, indicating the occurrence of UOR. The peak ≈500 cm^−1^ corresponding to the NiOOH characteristic peak increased with the rising voltage. As illustrated in Figure S4 (Supporting Information), UOR occurred preferentially in the presence of urea, which prompted NiOOH acted as the active species for UOR.^[^ ^47^ ^]^ The peak at 800 cm^−1^ attributing to Mo_2_C remained unchanged throughout the whole process. Additionally, in situ electrochemical Raman spectroscopy was conducted at 1.45 V to monitor the C─N bond state of urea during the UOR process. As shown in Figure S10 (Supporting Information), the characteristic C─N peak at 1000 cm^−1^ progressively decreased and eventually disappeared with prolonged reaction time, which indicated that urea underwent oxidation and finally degradation.

To further understand the electrocatalytic activity of Ni/Mo_2_C@CMF, density functional theory (DFT) calculations were performed based on the structure model of Ni (111) (Figure S11a, Supporting Information). First, the competitive adsorption of CO(NH_2_)_2_^*^ and OH^*^ on Ni (111) was investigated. As shown in Figure 3g, the adsorption energy of CO(NH_2_)_2_ on Ni was −1.156 eV, significantly lower than that of OH^*^ (−0.592 eV). Urea would preferentially occupy the active sites on Ni over OH^*^. The UOR mechanism was further elucidated by calculating the free energies of various reaction intermediates (Figures 3h). The dehydrogenation of the ^*^CONHN intermediate was the rate‐determining step (RDS) in the entire UOR process with a Gibbs free energy change (ΔG) of 1.13 eV.

### 2.3 HER Catalytic Activity

**Figure 4:**
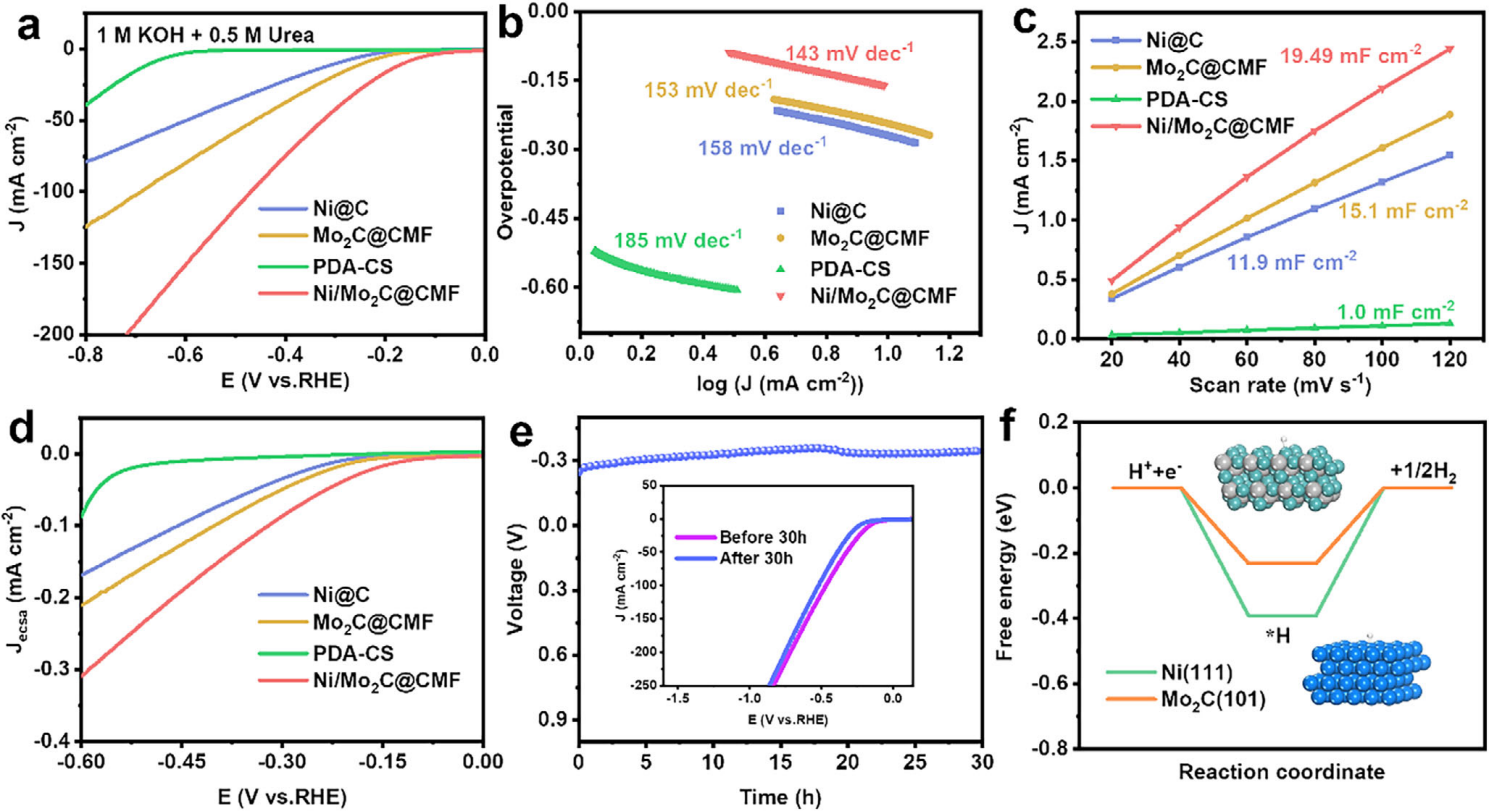
HER performance: a) LSV curves, b) Tafel curves, c) C_dl_, d) ECSA‐normalized LSV plots of Ni@C, Mo_2_C@CMF, PDA‐CS, and Ni/Mo_2_C@CMF, e) chronopotentiometry curve at the current density of 10 mA cm^−2^ (Inset: polarization curves before and after 30 h), f) calculated Gibbs free energy diagram.

For achieving the decoupling reaction of UOR/HER, a higher HER activity is also required over the Ni/Mo_2_C@CMF electrocatalyst. LSV curves for HER (**Figure** **4**a) demonstrated that Ni/Mo_2_C@CMF reached a current density of 10 mA cm^−2^ at only 163 mV, lower than PDA‐CS (669 mV), Mo_2_C@CMF (244 mV), and Ni@C (270 mV). In contrast, Ni@C and PDA‐CS exhibited poor HER activity, indicating the primary active role of Mo_2_C. The HER performance of Ni/Mo_2_C@CMF was also superior to Mo_2_C@CMF, showing the importance of Ni for enhancing its HER activity.^[^ ^43^ ^]^ Ni/Mo_2_C@CMF illustrated the smallest Tafel value of 143 mV dec^−1^ and the highest C_dl_ of 19.49 mF cm^−2^ (Figure 4b,c; Figure S12, Supporting Information). ECSA‐normalized LSV (Figure 4d) revealed the highest normalized current density of Ni/Mo_2_C@CMF. The time‐voltage curve at a current density of 10 mA cm^−2^ (Figure 4e) demonstrated only a minimal voltage shift within 30 h. Ni/Mo_2_C@CMF remained its structural integrity after the stability test (Figure S7a,c, Supporting Information). The model structure of Mo_2_C(101) was constructed in Figure S11b (Supporting Information). The calculated ΔG for activating HER on Ni(111) and Mo_2_C(101) was −0.39 and −0.23 eV, respectively (Figure 4f), which illustrated the lower energy barrier for HER on Mo_2_C than Ni.

### 2.4 Time Decoupled Metal‐Urea Battery

To estimate the feasibility of converting urea into hydrogen, a Zn–Urea battery was assembled with a bifunctional Ni/Mo_2_C@CMF catalyst as the cathode, a zinc foil as the anode, and an anion exchange membrane (AEM) as the separator (Figure 1). The anode electrolyte consisted of 1 Mm KOH/0.02 m Zn(CH_3_COO)_2_, and the cathode electrolyte contained 1 m KOH/0.5 m urea. During discharging, Zn was oxidized to Zn^2+^ at the anode while water was reduced to hydrogen at the cathode. In this process, the reduction potential of Zn was transferred to hydrogen, simultaneously generating electrical energy. During charging, urea oxidation occurred at the cathode, and Zn^2+^ at the anode was reduced back to Zn. In this process, external electrical energy was converted into the reduction potential of Zn and the oxidation potential of urea.

**Figure 5:**
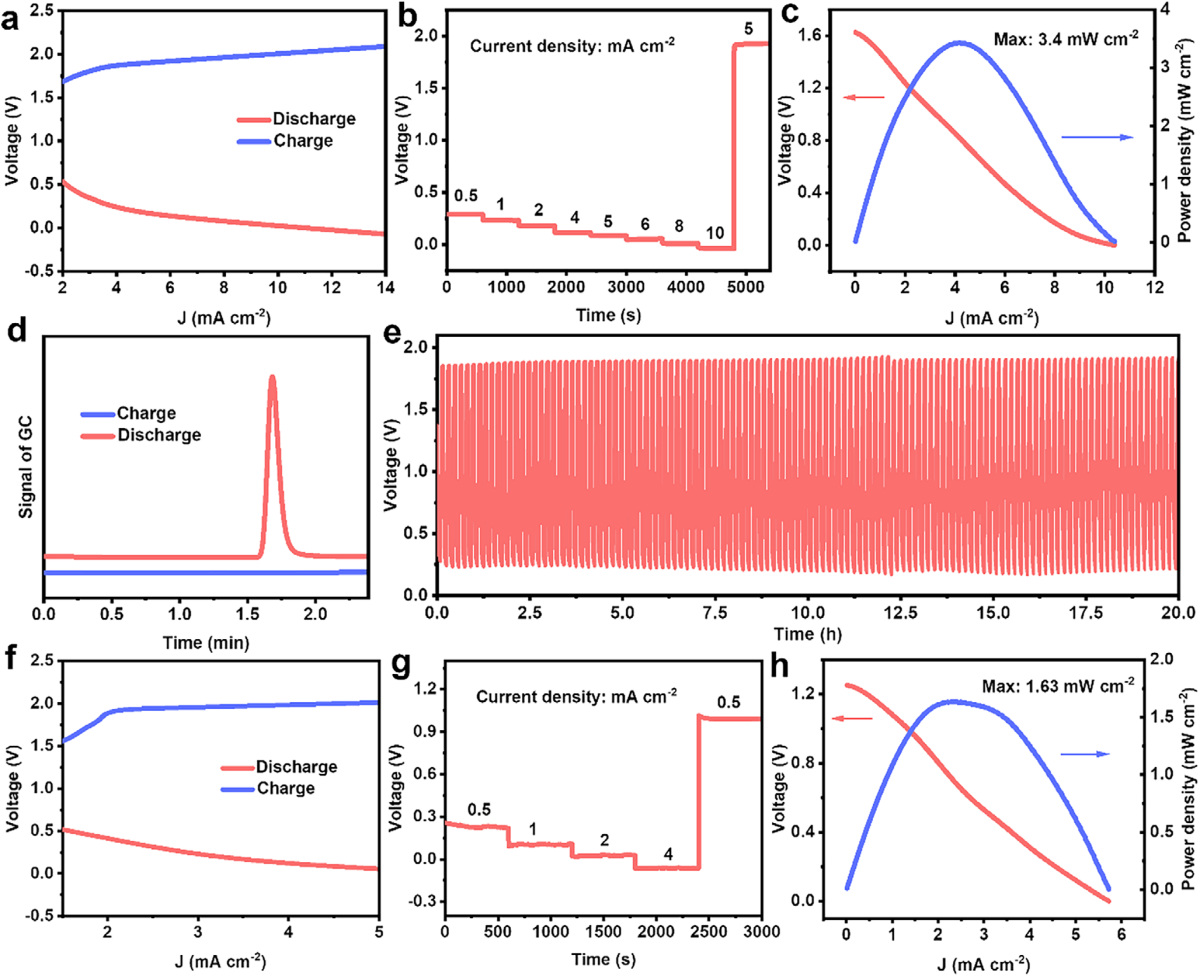
Performance of Zn–Urea battery: a) charge and discharge polarization curves, b) galvanostatic discharge/charge curves, c) discharging polarization (*j–V*) curve and the corresponding power density plot, d) gas chromatography during the discharge and charge processes at the current density of 5 mA cm^−2^ for 10 min, e) galvanostatic discharge–charge cycling curves at 5 mA cm^−2^. Performance of Zn–Urea battery Al–Urea battery: f) charge and discharge polarization curves, g) galvanostatic discharge/charge curves, h) discharging polarization (*j–V*) curve, and the corresponding power density plot.

The Zn–Urea battery efficiency was evidenced by the fact that the open circuit voltage of 1.27 V remained stable for more than 60 min (Figure S13, Supporting Information). In **Figure** **5**a, the charge–discharge polarization curves during the discharge and charge processes further demonstrate the practicality of Zn–Urea battery. The rechargeability of Zn–Urea battery was verified by the galvanostatic discharge/charge curves in Figure 5b, which were tested at various discharge current densities from 0.5 to 10 mA cm^−2^ and subsequently charged at 5 mA cm^−2^ after continuous discharging. Additionally, the maximal power density of Zn–Urea battery reached 3.4 mW cm^−2^, exceeding the majority of aqueous metal‐redox bi‐catalyst batteries in previous research (Figure 5c; Table S1, Supporting Information). As shown in Figure 5d, no hydrogen production was observed after charging at a current density of 5 mA cm^−2^ for 10 min. Nevertheless, upon discharging at the same current density for 10 min, a substantial quantity of hydrogen was detected. The hydrogen production rate was ≈0.092 mmol h^−1^ cm^−2^ with calculated Faradaic efficiency (FE) of 99%, which was higher than other batteries for hydrogen production (Table S1, Supporting Information). As illustrated in Figure 5e, the Zn–Urea battery exhibited excellent stability over 20 h at a current density of 5 mA cm^−2^ with no significant voltage fluctuation.

To verify the universality of this battery device, an Al–Urea battery was also assembled and tested (Figure S14a, Supporting Information). The reaction in cathode was similar to Zn–Urea battery but difference in the anode. During discharging, Al was oxidized to Al^3+^ at the anode, and the reduction potential of Al was transferred to hydrogen, simultaneously generating the electrical energy. As shown in Figure S14b (Supporting Information), the open circuit voltage of 1.38 V remained stable for 60 min. And the open circuit voltage was higher than Zn–Urea battery, because the theoretically electromotive voltage of Al (Al^3+^+3e^−^ = Al, −1.662 V vs NHE at pH 0) is higher than Zn (Zn^2+^+2e^−^ = Zn, −0.76 V vs NHE at pH 0).^[^ ^48^ ^]^ The full‐cell voltage difference of 0.11 V in two batteries was smaller than the theoretical anode potential gap of ≈0.905 V. This difference might result from the significant difference in the solubility products (K_sp_) of metal hydroxides in alkaline electrolytes. Al(OH)_3_ has a lower K_sp_ (1.3 × 10^−33^) than Zn(OH)_2_ (3 × 10^−17^). As a result, part of Al would form Al(OH)_3_ during cell operation and deposit on the anode. The deposited Al(OH)_3_ might increase the anode polarization, which would result in a reduced full‐cell voltage. The charge–discharge curves in Figure 5f highlighted the practical applicability of the Al–Urea battery. The galvanostatic discharge/charge curves tested at various discharge current densities from 0.5 to 4 mA cm^−2^ and then charging at 0.5 mA cm^−2^ (Figure 5g) confirmed the rechargeability of the Al–Urea battery. Moreover, the peak power density of the battery reached 1.6 mW cm^−2^ (Figure 5h). The lower power density might be attributed to the increased anode polarization caused by the deposited Al(OH)_3_. Similarly, a significant amount of hydrogen was detected upon discharging under the current density of 2 mA cm^−2^ for 10 min (Figure S14c, Supporting Information). The hydrogen production rate was ≈0.037 mmol h^−1^ cm^−2^ with a FE value of 99%. At a current density of 1 mA cm^−2^, the Al–Urea battery demonstrated excellent stability over 10 h (Figure S14d, Supporting Information). In addition, the Mg–Urea battery was also assembled and tested. Its battery performance was poor compared with Zn and Al–Urea batteries (Figure S15, Supporting Information), which may be resulted from the inherent inertness of Mg in alkaline solutions.

### 2.5 Time Decoupled Zn–Urine Battery

**Figure 6:**
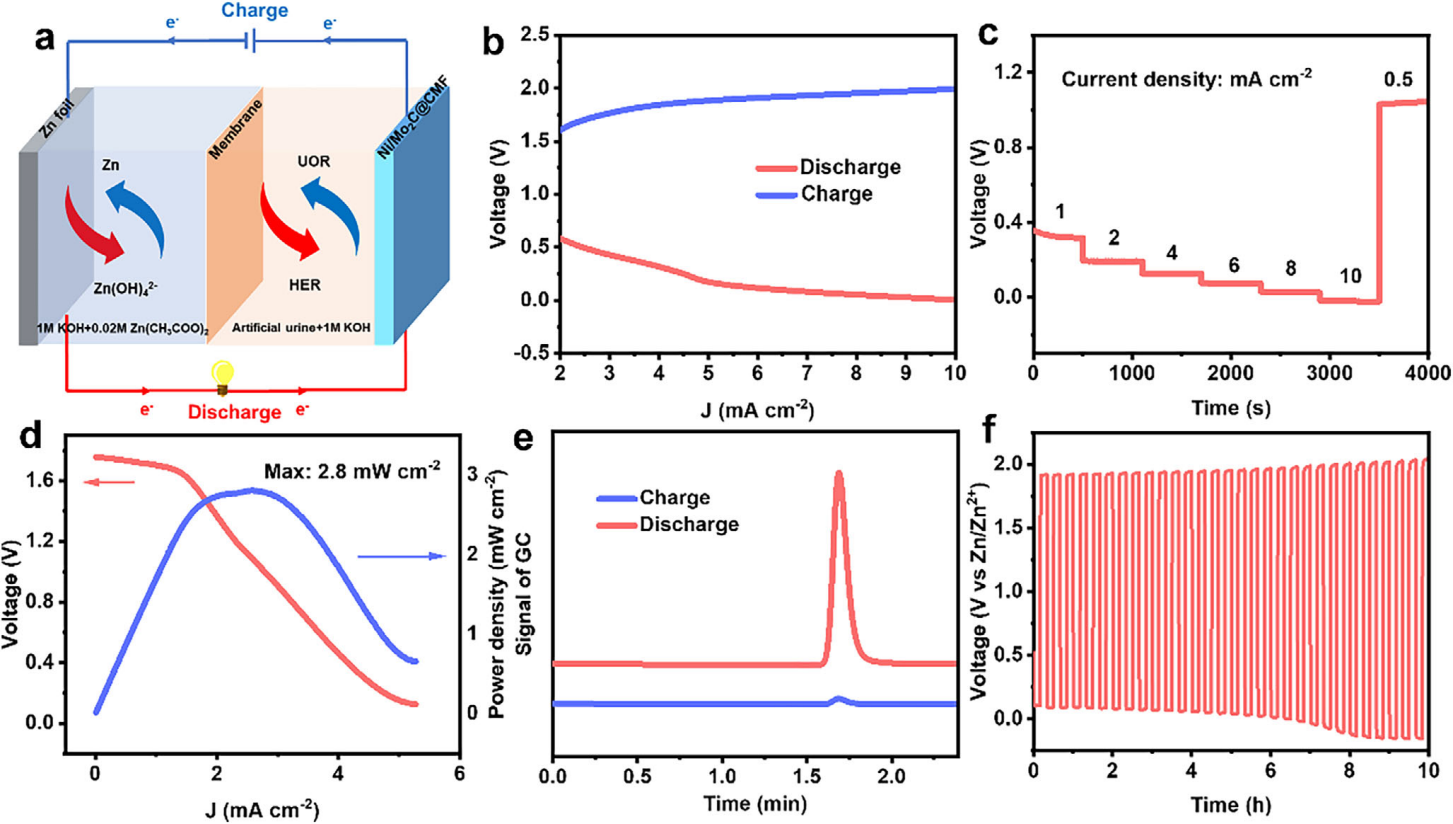
a) Schematic illustration of Zn–Urine battery. Performance of Zn–Urine battery: b) charge and discharge polarization curves, c) galvanostatic discharge/charge curves, d) discharging polarization (*j–V*) curve and the corresponding power density plot, e) gas chromatography during discharge and charge processes at the current density of 3 mA cm^−2^ for 10 min, f) galvanostatic discharge–charge cycling curves at 5 mA cm^−2^.

To assess the practical application potential of this battery system for decoupled hydrogen production from urea wastewater, a Zn–Urine battery was assembled and tested. As shown **Figure** **6**a, the anode and cathode were similar to Zn–Urea battery, but the cathode electrolyte was changed to artificial urine (urea concentration: 20 g L^−1^) with 1 m KOH. As shown in Figure S16 (Supporting Information), the open‐circuit voltage remained stable at 1.26 V for over 60 min. The charge–discharge polarization curves in Figure 6b highlighted the practical applicability of Zn–Urine battery. Figure 6c presents the constant‐current discharge/charge curves tested at various discharge current densities ranging from 1 to 10 mA cm^−2^. After continuous discharge, the battery was recharged at 0.5 mA cm^−2^. The peak power density of the battery reached 2.8 mW cm^−2^ (Figure 6d), slightly lower than Zn–Urea battery. The significant quantity of hydrogen was also detected during the same time frame of discharging at the current density of 3 mA cm^−2^ (Figure 6e). The hydrogen production rate was 0.055 mmol h^−1^ cm^−2^ with FE of 98%. The Zn–Urine battery could operate stably for 7 h (Figure 6f). The performance of the battery exhibited a relatively rapid decline after 7 h, in which complex components in urine (e.g., uric acid, creatinine) might lead to a deterioration of the catalyst's performance.

## 3 Conclusion

In summary, a temporally decoupled rechargeable metal‐urea battery has been developed for the conversion of urea into pure hydrogen. By overcoming the kinetic mismatch constraints of conventional electrolysis based on bifunctional Ni/Mo_2_C@CMF electrocatalysts, this innovative approach finally achieved the continuous and efficient conversion of urea to hydrogen in home‐made Zn–Urea, Al–Urea, and Zn–Urine batteries. In particular, the Zn–Urea battery enabled effective urea‐to‐hydrogen conversion with a Faraday efficiency of 99% and continuous hydrogen production over 20 h with a maximum power density of 3.4 mW cm^−2^. The reported decoupled electrocatalytic mechanism with a dual‐functional catalyst offers an impressive route for energy collection and resource utilization of urea wastewater.

## Conflict of Interest

The authors declare no conflict of interest.

## Supporting information



Supporting Information

## Data Availability

The data that support the findings of this study are available from the corresponding author upon reasonable request.
