# Supplementary material for: Time‐Decoupled Electrolysis via a Rechargeable Metal‐Urea Battery for Waste Urea Treatment and Hydrogen Production
Source: Adv Sci (Weinh). 2025 Jul 17;12(38):e07657. doi: 10.1002/advs.202507657 (PMC12520516; doi:10.1002/advs.202507657)
Supplement: Supplementary file 1 — Supporting Information [file ADVS-12-e07657-s001.docx]

**Supporting Information**

**Time-Decoupled Electrolysis *via* a Rechargeable Metal-Urea Battery for Waste Urea Treatment and Hydrogen Production**

*Dong Lv, Zhengrong Xu, Xingyu Guo, Deng Liu and Rui Liu**

Key Laboratory of Advanced Civil Engineering Materials of Ministry of Education, School of Materials Science and Engineering, Tongji University, Shanghai 201804, China

*Corresponding author: ruiliu@tongji.edu.cn

**Experimental Section**

**1. Materials**

Ammonium molybdate tetrahydrate ((NH_4_)_6_Mo_7_O_24_·4H_2_O) and dopamine hydrochloride were purchased from Aladdin Co., Ltd. Nickel nitrate hexahydrate (Ni(NO_3_)_2_·6H_2_O), ethanol and ammonia solution (NH_3_·H_2_O) were purchased from Sinopharm Chemical Reagent Co., Ltd. Nafion (5.0 wt.%) was purchased from Shanghai Hesen Electric Co., Ltd. All chemicals were purchased commercially and used without further purification.

**2. Preparation of Ni/Mo_2_C@CMF, Mo_2_C@CMF, Ni@C and PDA-CS**

707 mg of (NH_4_)_6_Mo_7_O_24_·4H_2_O and 582 mg of Ni(NO_3_)_2_·6H_2_O were first dissolved in 36 mL of water and 16 mL of ethanol. Then, 0.6 mL of NH_3_·H_2_O was added to adjust solution pH to ~8. Next, 20 mL of dopamine solution (20 mg·mL^-1^) was dropwise added to the above solution, and the mixture was continuously stirred at room temperature overnight. The solid Ni/Mo-PDA precursor was collected by centrifugation and washed repeatedly with water and ethanol. Then, the Ni/Mo-PDA precursor was annealed at 750 °C under N_2_ for 3 h with a heating rate at 2 °C min^-1^ to obtain Ni/Mo_2_C@CMF. Control samples Mo_2_C@CMF, Ni@C and PDA-CS were synthesized following the same procedure except the absence of Ni(NO_3_)_2_·6H_2_O, (NH_4_)_6_Mo_7_O_24_·4H_2_O, both (NH_4_)_6_Mo_7_O_24_·4H_2_O and Ni(NO_3_)_2_·6H_2_O, respectively.

**3. Characterization**

The morphology and structure were observed using transmission electron microscope (TEM) (FEI Talos F200x) and scanning electron microscope (SEM) (ZEISS Sigma 300). The crystallographic structure and phase composition were characterized by X-ray diffraction spectrometry (XRD, D8 ADVANCE) with Cu Kα radiation. X-ray photoelectron spectroscopy (XPS, Thermo Scientific K-Alpha) was utilized to investigate the composition and chemical state. *In-situ* Raman spectroscopy (Horiba LabRAM Odyssey) data were obtained with a laser wavelength of 532 nm.

**4. Electrochemical measurements**

The electrochemical measurements were carried out using a CHI 760E electrochemistry workstation (Chen Hua Instruments Co., China) at room temperature. The three-electrode system consisted of working electrode (carbon paper loaded with catalyst, area of 1 cm^2^), reference electrode (Hg/HgO, 1 M KOH), and counter electrode (graphite rod). The electrocatalyst homogeneous ink was typically prepared as follows: 10 mg catalyst was dispersed in 1 mL ethanol solution and 30 μL of 5.0 wt.% Nafion solution, and then sonicated for 30 min. Subsequently, the dispersion (100 μL) was casted on carbon paper (∼1 mg cm^-2^). The deposited catalyst film was dried at room temperature. Before the catalytic test, the catalyst was activated through cyclic voltammetry (CV) test in the potential range from 0 to 1.7 V *vs*. RHE at the scan rate of 10 mV s^-1^ for 5 cycles. All the linear sweep voltammetry (LSV) measurements were recorded at a scan rate of 5 mV s^-1^ in 1 M KOH with 0.5 M urea, and the data were presented without iR correction. All the potentials were converted to reversible hydrogen electrode (RHE) (E_RHE_ = E_Hg/HgO_ + 0.059 × pH + 0.098). Tafel slope was calculated according to the equation as follows: η = a + b × log j$\text{η = a + b × log j}$, where η, a, b, and j are the overpotential, constant, Tafel slope, and current density, respectively. Electrochemically active area (ECSA) was evaluated by double layer capacitance (C_dl_) measured *via* CV at different scan rates. Electrochemical impedance spectroscopy (EIS) was performed with a frequency scan range from 0.1 Hz to 1 MHz.

**5. Theoretical calculations**

Density functional theory (DFT) calculations were performed by the projector augmented wave (PAW) method.^[1, 2]^ The PAW pseudopotential with the Perdew-Burke-Ernzerhof (PBE) generalized gradient approximation (GGA) exchange correlation function was utilized in the computations.^[3-5]^ The cutoff energy of the plane waves basis set was 500 eV and a Monkhorst-Pack mesh of 2×2×1 was used in K-sampling. All structures were spin polarized and all atoms were fully relaxed with the energy convergence tolerance of 10^-5^ eV per atom, and the final force on each atom was < 0.05 eV Å^-1^. Finally, the adsorption energies (E_ads_) were calculated as E_ads_= E_ad/sub_ -E_ad_ -E_sub_, where E_ad/sub_, E_ad_, and E_sub_ are the total energies of the optimized adsorbate/substrate system, the adsorbate in the structure, and the clean substrate, respectively. The free energy was calculated using the equation: G=E_ads_+ZPE-TS, where G, E_ads_, ZPE and TS are the free energy, total energy from DFT calculations, zero point energy and entropic contributions, respectively.

**6. Metal-Urea battery measurement**

**Zn-Urea battery:** The battery was assembled with a bifunctional Ni/Mo_2_C@CMF catalyst as the cathode, a zinc foil as the anode, and an anion exchange membrane (AEM) as the separator. The anode electrolyte consisted of 1 M KOH/0.02 M Zn(CH_3_COO)_2_ and the cathode electrolyte contained 1 M KOH/0.5 M urea.

**Al-Urea battery:** The battery was assembled with a bifunctional Ni/Mo_2_C@CMF catalyst as the cathode, an Al foil as the anode, and an anion exchange membrane (AEM) as the separator. The anode electrolyte consisted of 1 M KOH/0.02 M AlCl_3_, and the cathode electrolyte contained 1 M KOH/0.5 M urea.

**Mg-Urea battery:** The battery was assembled with a bifunctional Ni/Mo_2_C@CMF catalyst as the cathode, a Mg foil as the anode, and an anion exchange membrane (AEM) as the separator. The anode electrolyte consisted of 1 M KOH/0.02 M MgSO_4_, and the cathode electrolyte contained 1 M KOH/0.5 M urea.

**Zn-Urine battery:** The battery was assembled with a bifunctional Ni/Mo_2_C@CMF catalyst as the cathode, a zinc foil as the anode, and an anion exchange membrane (AEM) as the separator. The anode electrolyte consisted of 1 M KOH/0.02 M Zn(CH_3_COO)_2_, and the cathode electrolyte contained 1 M KOH and artificial urine (urea concentration: 20 g L^-1^).

**H_2_ Faraday efficiency (FE) test:**

Gas products were quantified by gas chromatography (GC-2014C, SHIMADZU), which was equipped with the thermal conductivity detector (TCD) for online analysis. Argon was selected as carrier gas. Faradaic efficiency of gas product was calculated by the formula:

$$FE\left( \% \right)=\frac{v(vol\%)\times V\times N\times F\times p}{R\times T\times i_{total}}$$

where $v(vol\%)$ represents the volume concentration of gas detected by GC, $V$ is the flow rate of gas injection, $N$ is the number of electron transfers, *F*, *p*, *R* and *T* are Faraday constant (96485 C mol^−1^), standard atmosphere (101.325 kPa), gas constant (8.314 J mol^−1^ K^−1^) and room temperature (298.15 K), respectively. $i_{total}$ is the total current at applied potential.

Hydrogen production rate ($v$) calculated by the formula:

$$v=\frac{n}{t\times A}$$

Where $n$ represents the amount of substance of hydrogen (mol), $t$ and $A$ are time (h) and cathode active area (cm^-2^).


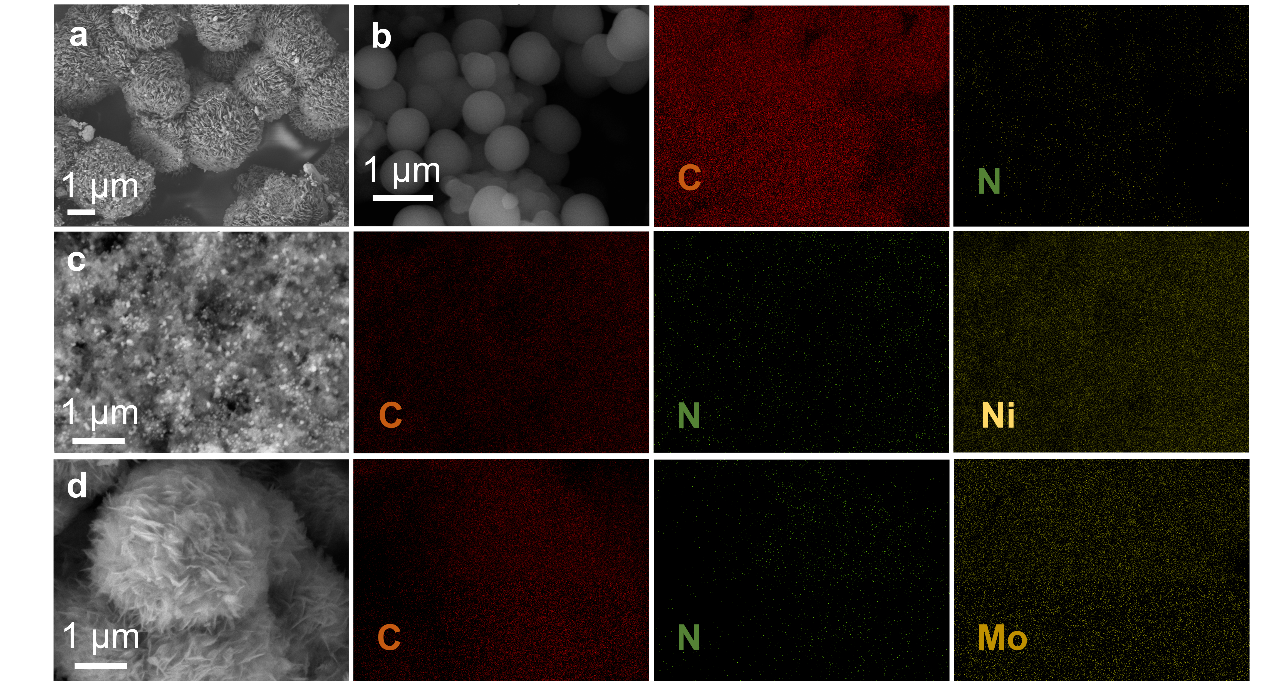


Figure S1. SEM image of (a) Ni/Mo_2_C@CMF. SEM and EDS mapping images of (b) PDA-CS, (c) Ni@C and (d) Mo_2_C@CMF.


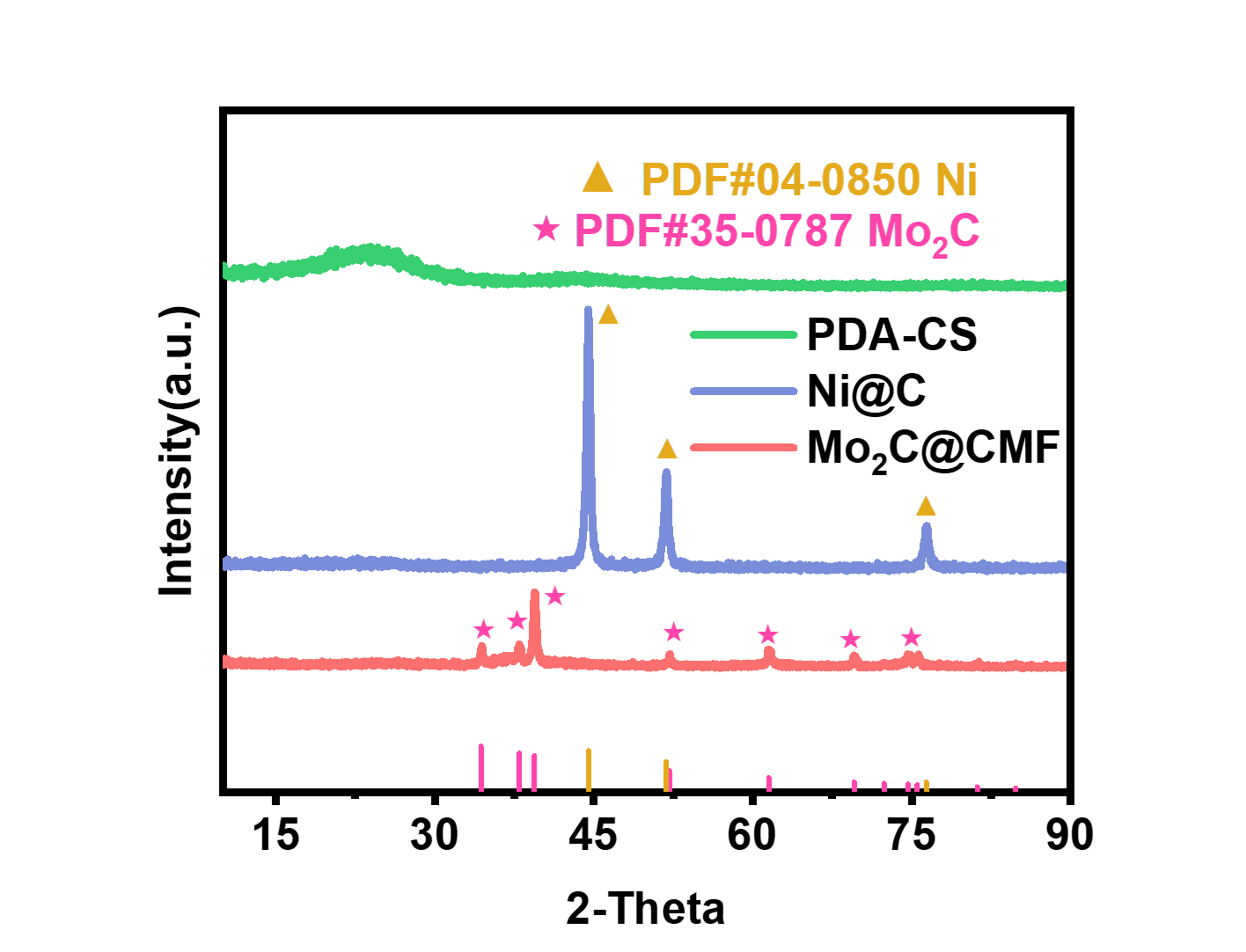


Figure S2. XRD patterns of PDA-CS, Ni@C and Mo_2_C@CMF.


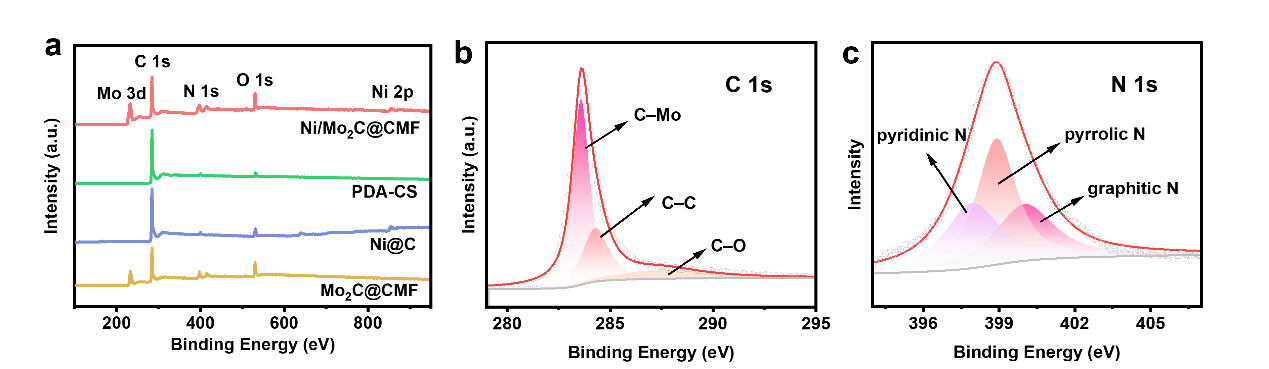


Figure S3. (a) XPS full spectra of samples, (b) C 1s and (c) N 1s XPS spectra of Ni/Mo_2_C@CMF.


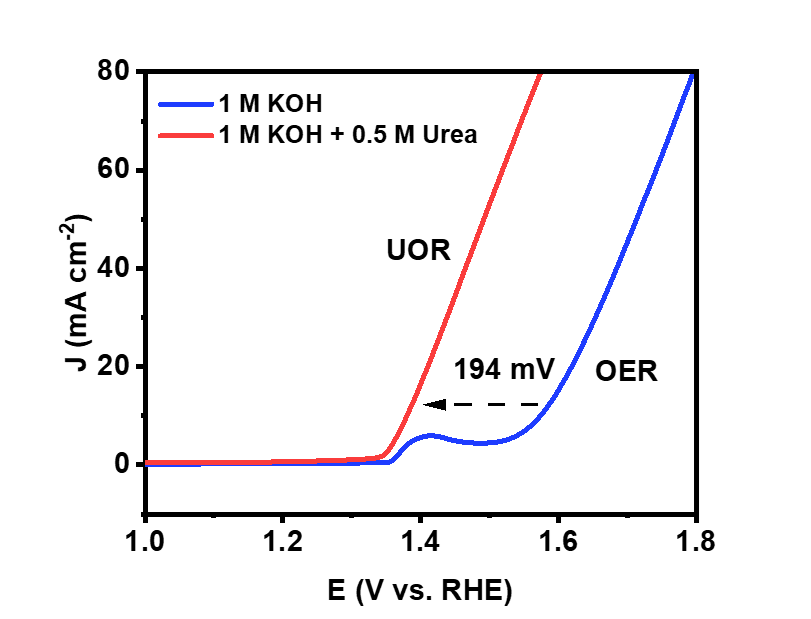


Figure S4. LSV curves of Ni/Mo_2_C@CMF in different electrolytes.


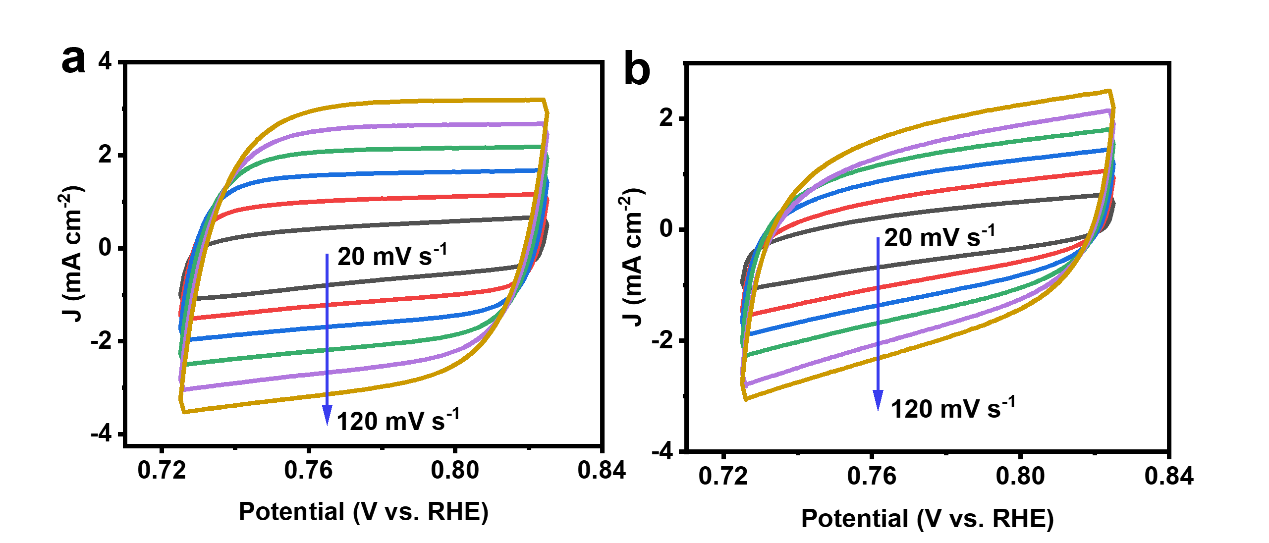


Figure S5. CV curves of (a) Ni/Mo_2_C@CMF and (b) Ni@C tested at various scan rates from 20 to 120 mV s^-1^ in the potential range of 0.725-0.825 V.


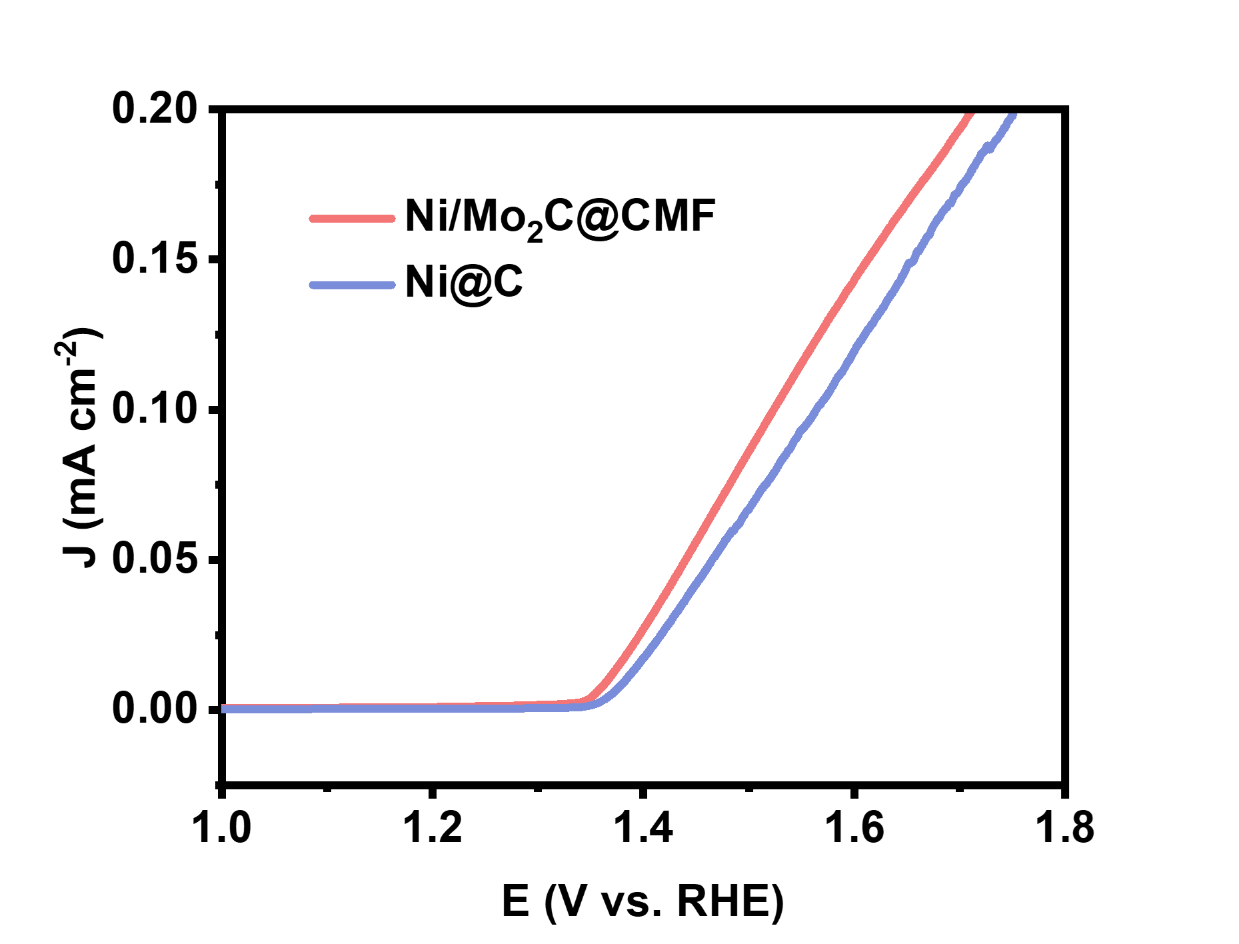


Figure S6. ECSA-normalized LSV plots of Ni/Mo_2_C@CMF and Ni@C for UOR.


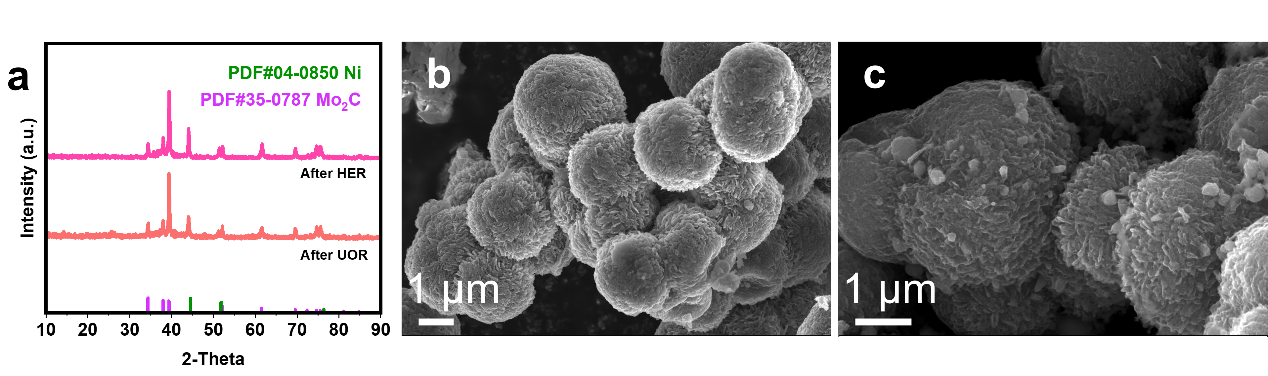


Figure S7. (a) XRD patterns of Ni/Mo_2_C@CMF after UOR and HER; SEM images after (b) UOR and (c) HER.

Figure S8. Raman spectra of Ni/Mo_2_C@CMF with and without the presence of urea.


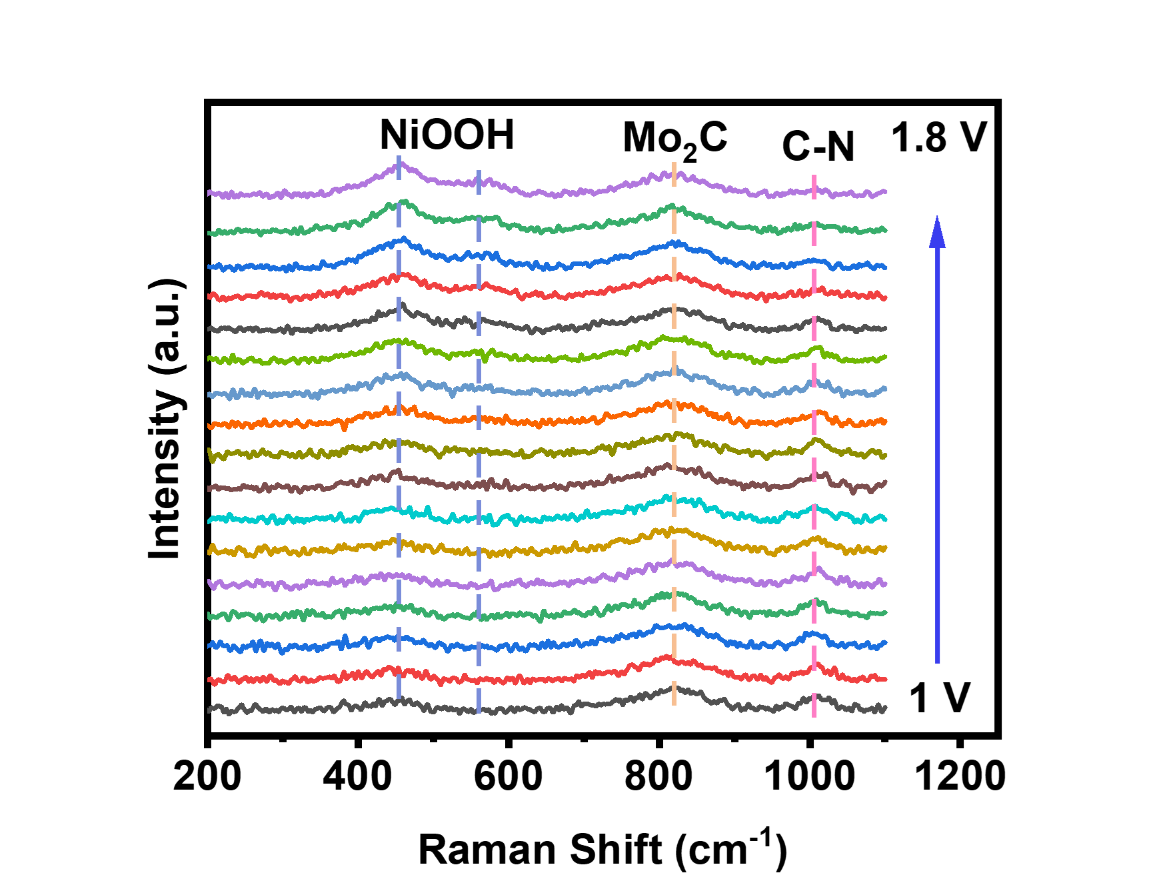

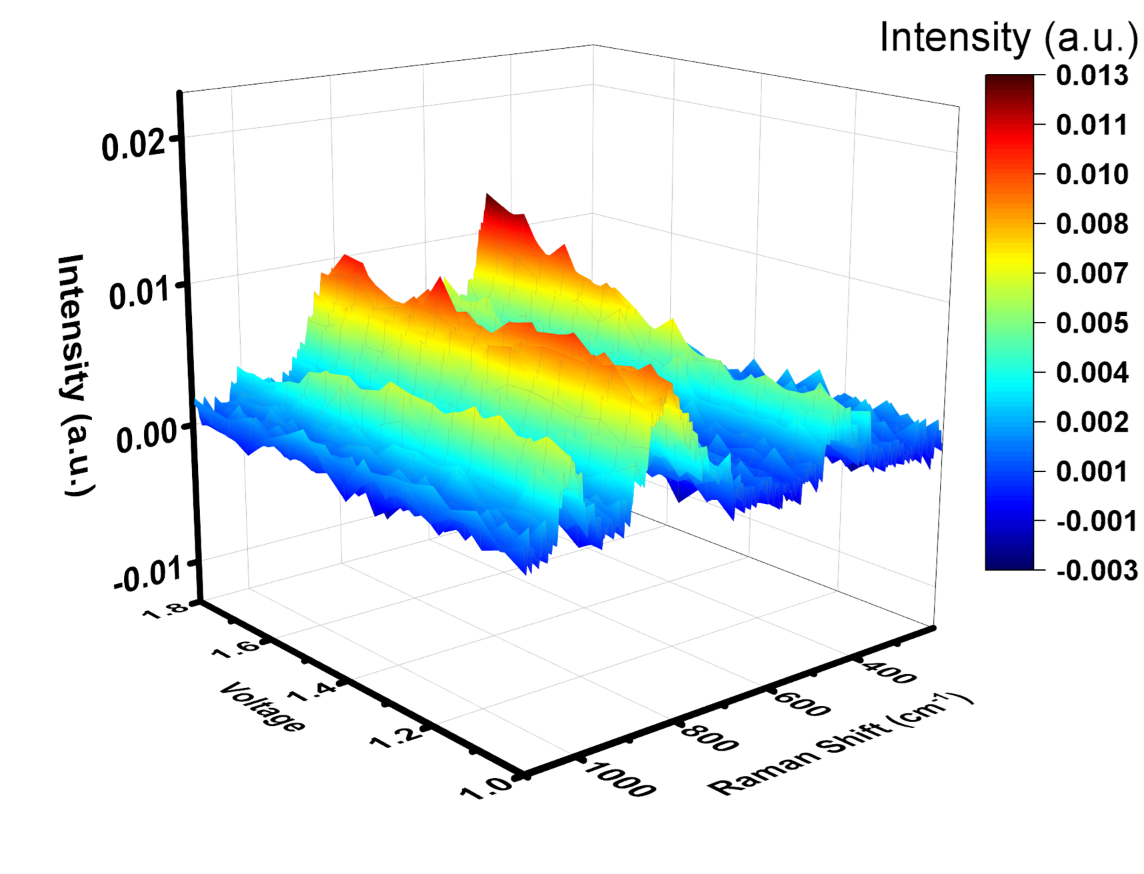


**b**

**a**

Figure S9. Potential-dependent in situ Raman spectra of Ni/Mo_2_C@CMF during the potential range from 1.0 to 1.8 V: (a) two-dimensional graph and (b) three-dimensional graph.


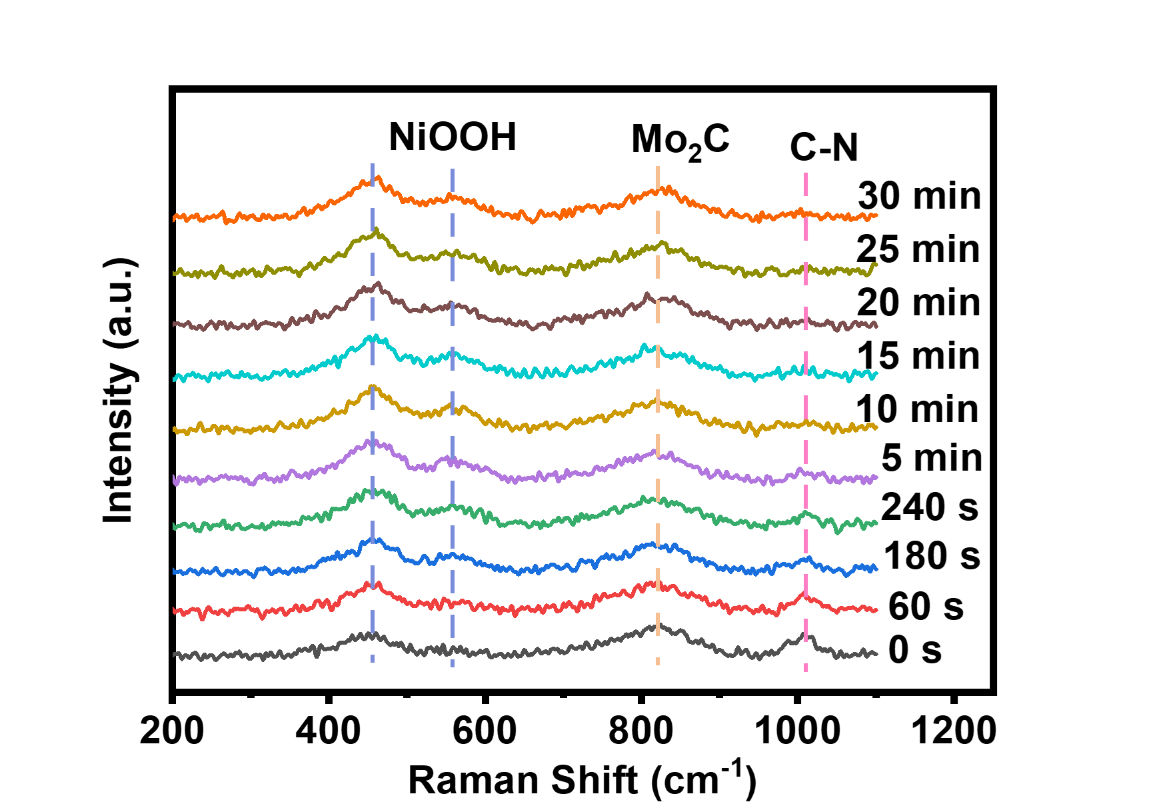

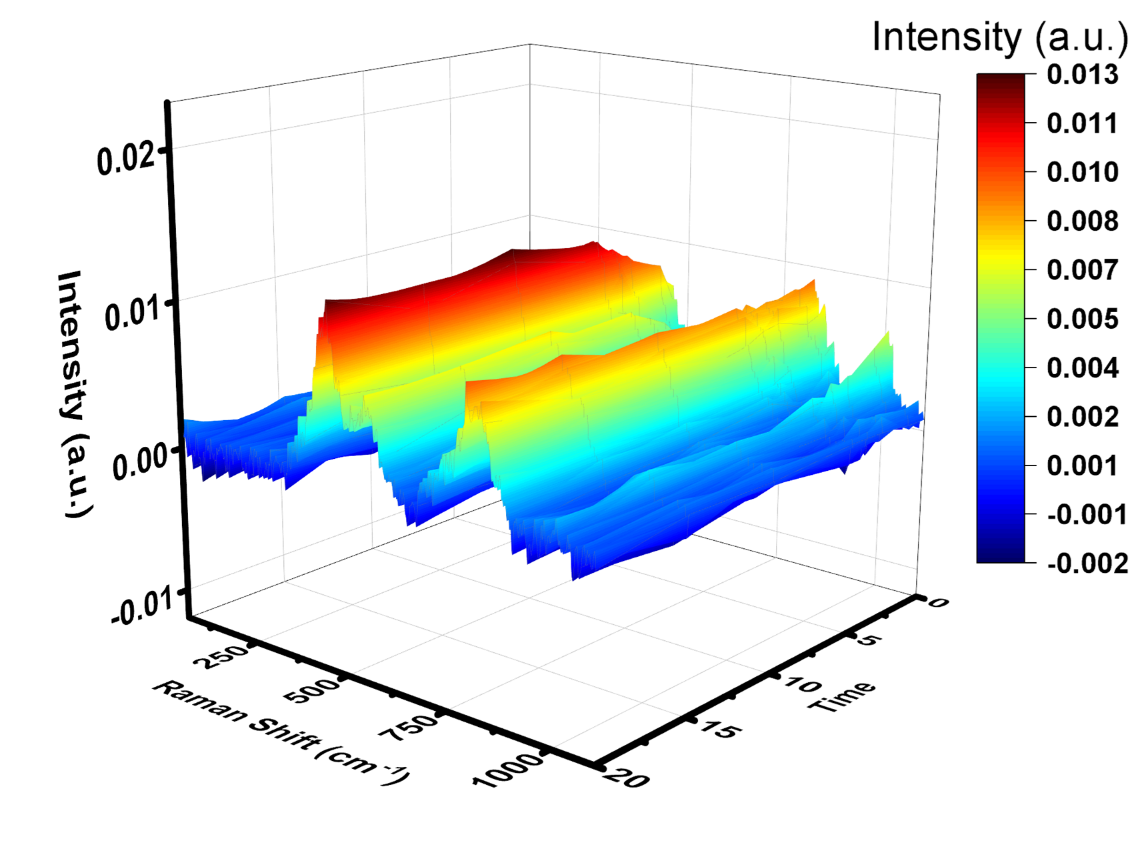


**b**

**a**

Figure S10. Time-dependent in situ Raman spectra of Ni/Mo_2_C@CMF during the potential range from 0 to 30 min: (a) two-dimensional graph and (b) three-dimensional graph.


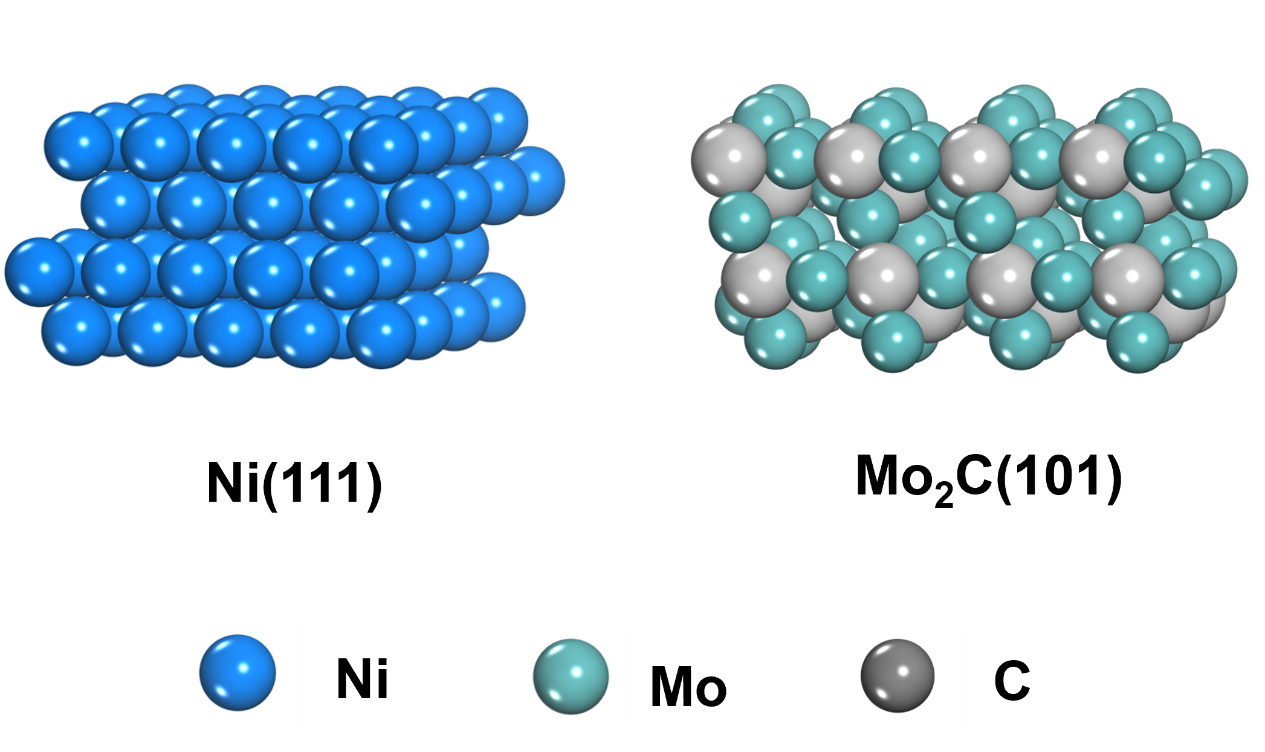


**b**

**a**

Figure S11. Structure models of (a) Ni (111) and (b) Mo_2_C (101).


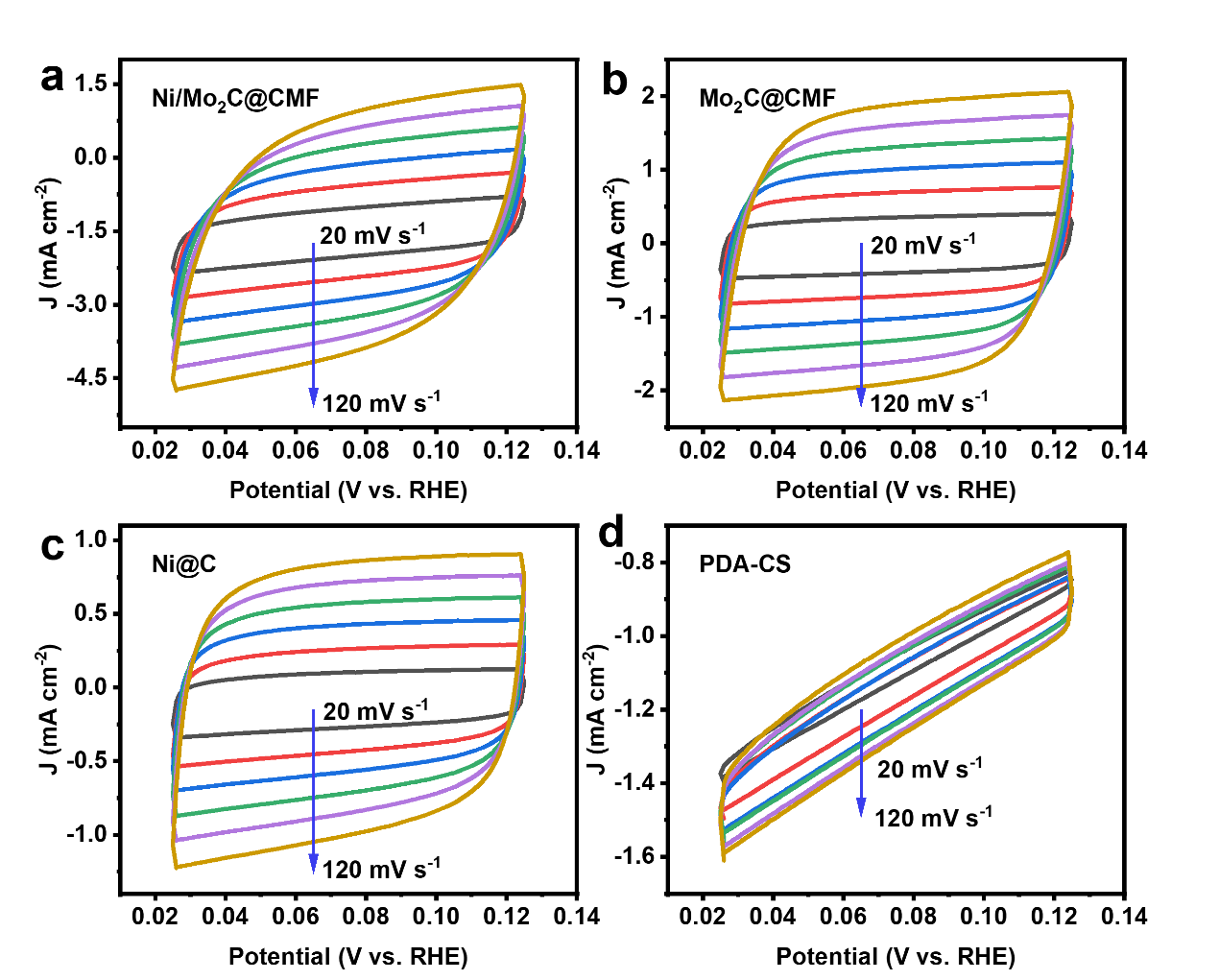


Figure S12. CV curves of (a) Ni/Mo_2_C@CMF, (b) Mo_2_C@CMF, (c) Ni@C and (d) PDA-CS tested at various scan rates from 20 to 120 mV s^-1^ in the potential range of 0.026-0.126 V.


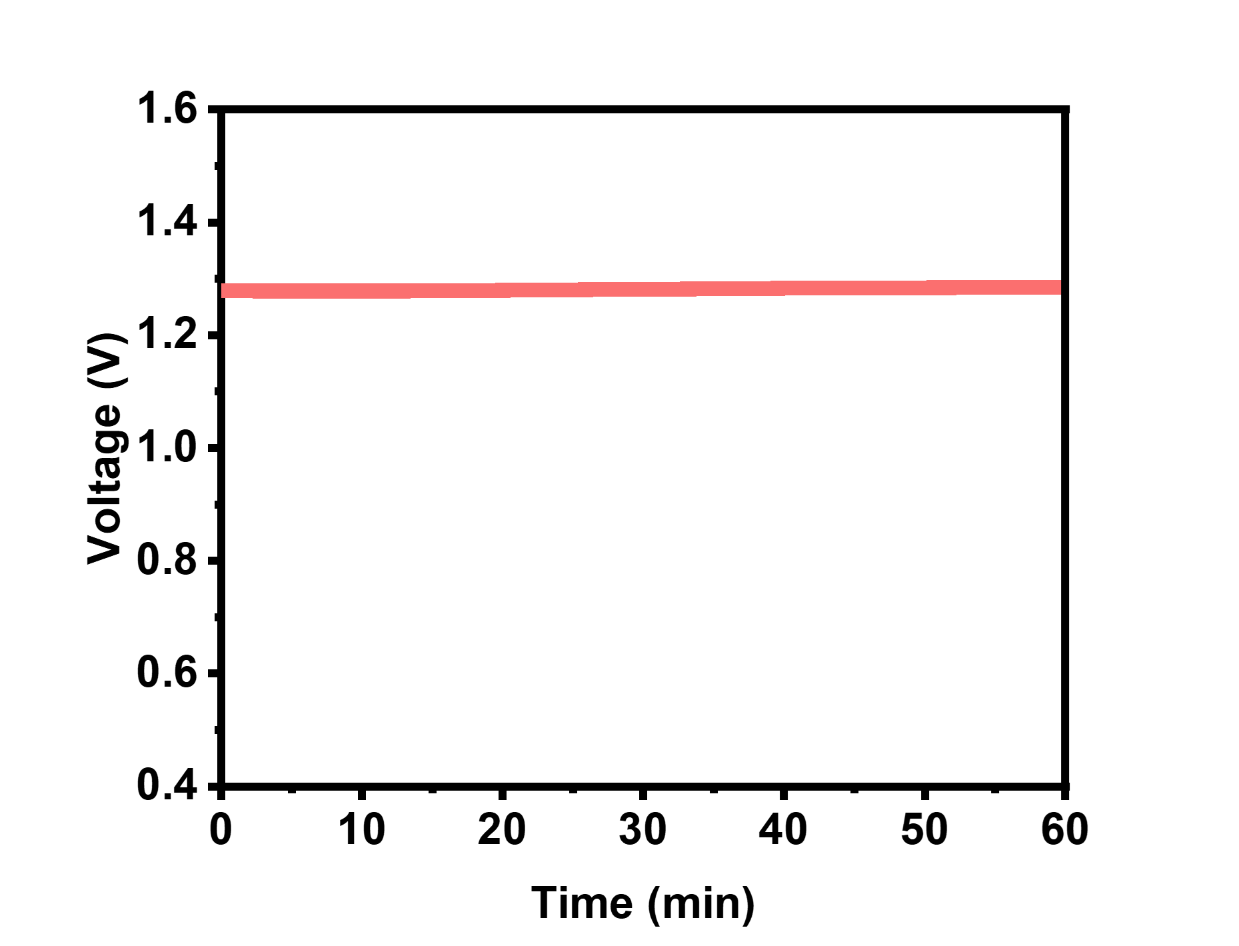


Figure S13. Open circuit voltage of Zn-Urea battery for 60 min.


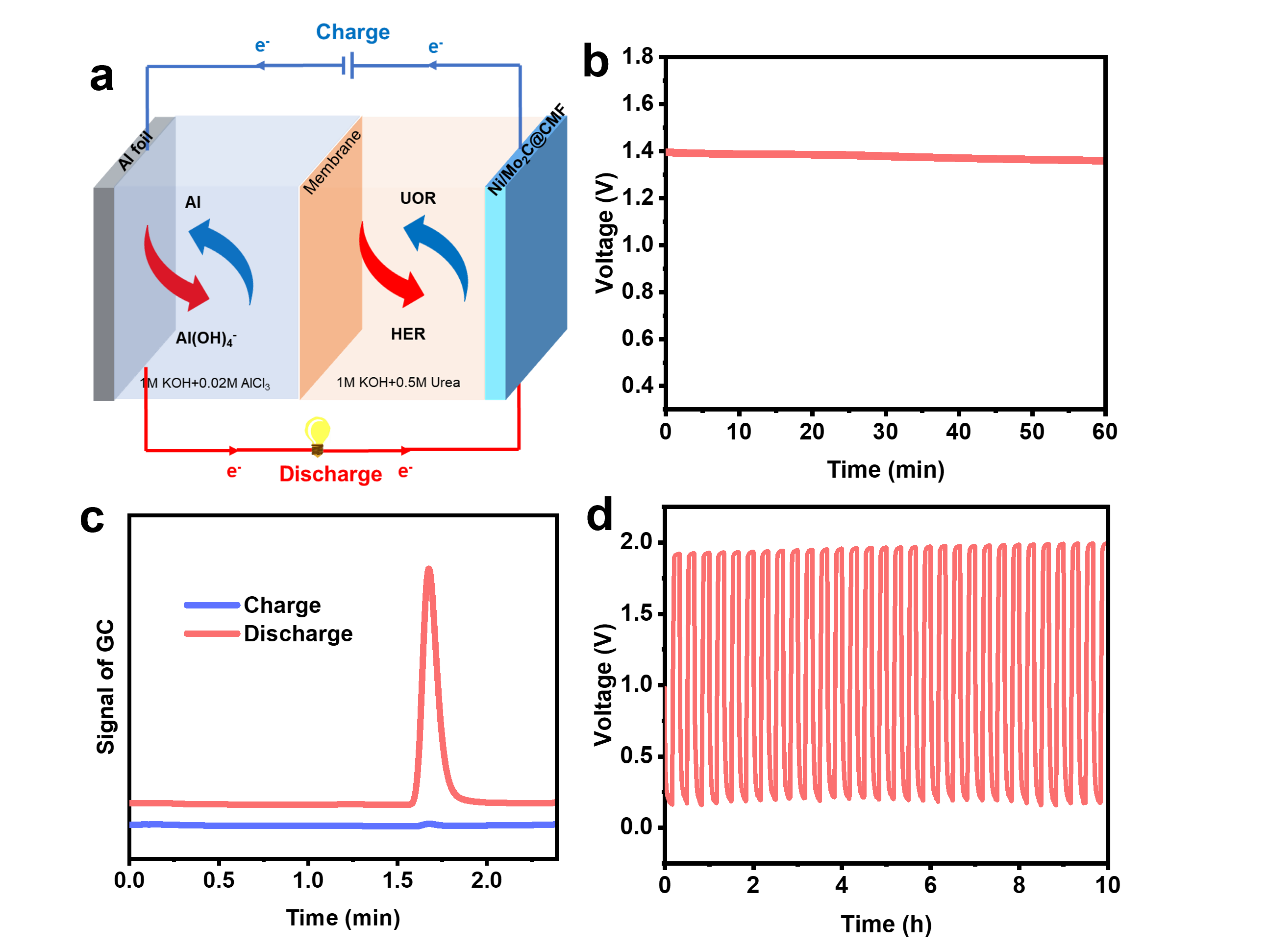


Figure S14. (a) Schematic illustration of Al–Urea battery. (b) Open circuit voltage of Al-Urea battery for 60 min. (c) Gas chromatography during discharge and charge processes at the current density of 2 mA cm^-2^ for 10 min. (d) Galvanostatic discharge–charge cycling curves of Al-Urea battery at 1 mA cm^-2^.


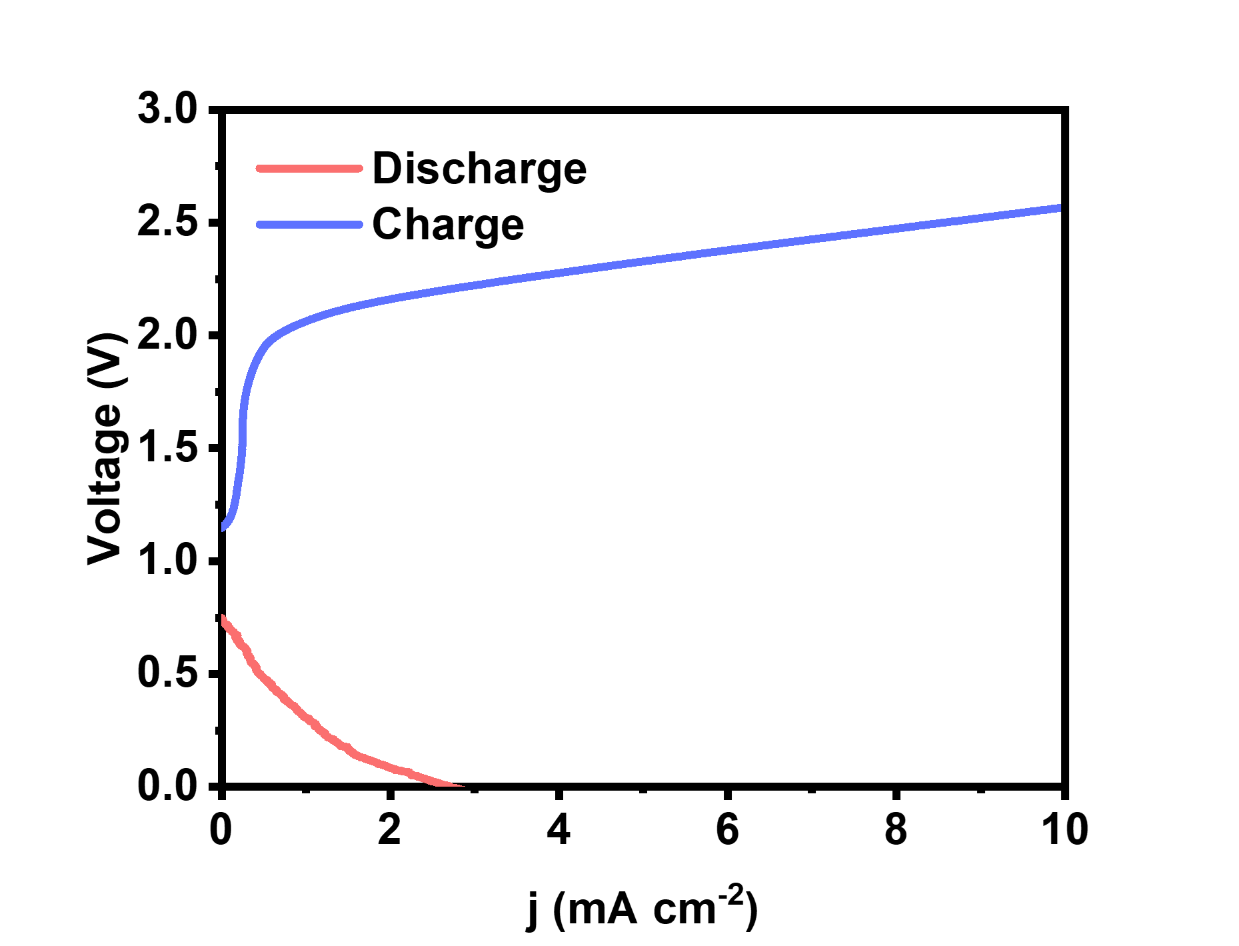


Figure S15. Charge and discharge polarization curves of Mg-Urea battery.


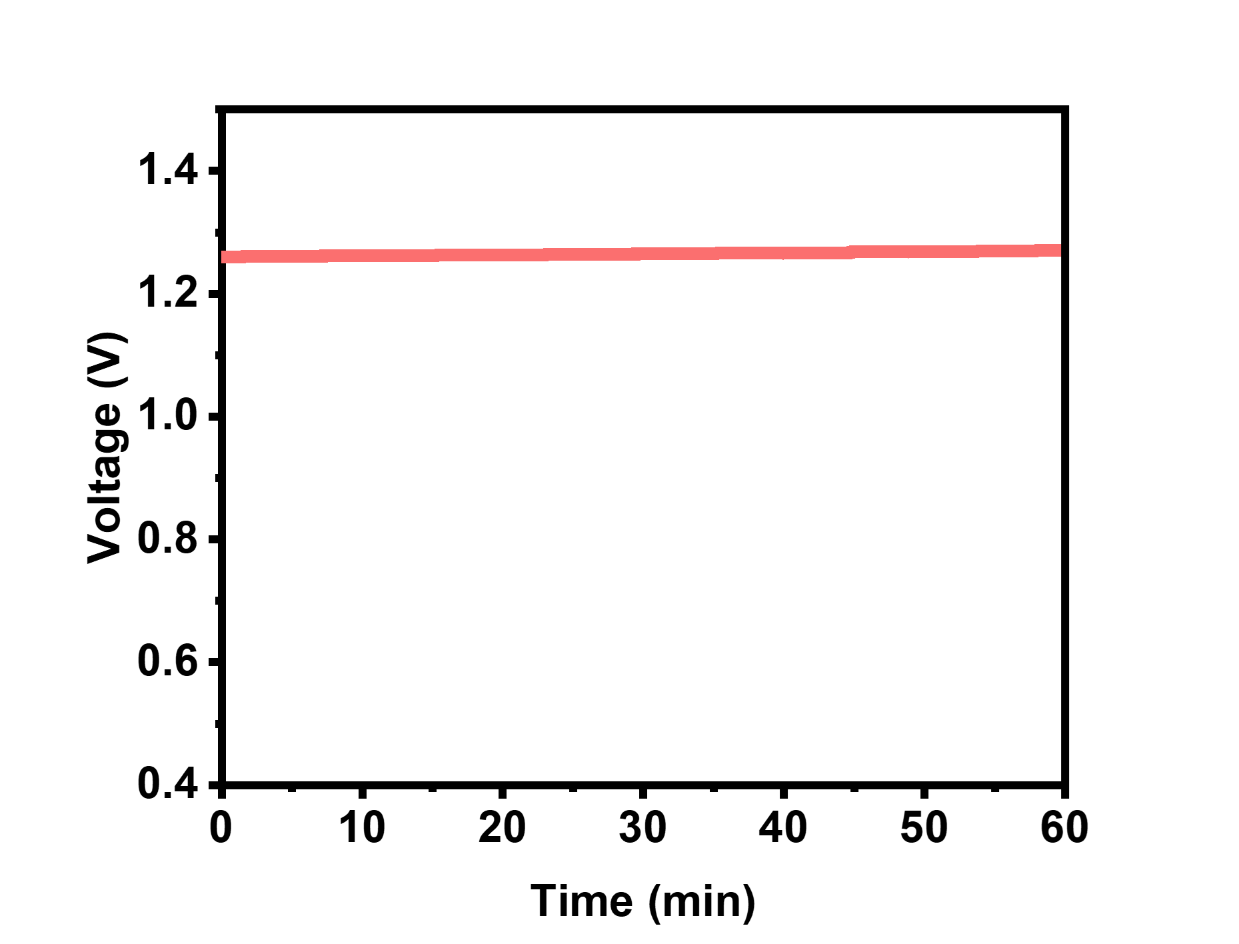


Figure S16. Open circuit voltage of Zn-Urine battery for 60 min.

Table S1. Comparison with other aqueous metal-redox double catalyst batteries.

| Cathode Catalyst | Battery | Product | Maximum power density (mW cm^-2^) | Faraday efficiency (%) | Ref. |
| --- | --- | --- | --- | --- | --- |
| 3D porous Pd | Zn-CO_2_ | HCOOH | __ | 80 | ^[6]^ |
| Ir@Au | Zn-CO_2_ | CO | __ | 67 | ^[7]^ |
| s-PdNi | Zn-CO_2_ | CO | 1.95 | 92.6 | ^[8]^ |
| Ni@N-C | Zn-CO_2_ | CO | 1.64 | 90 | ^[9]^ |
| Fe_1_-Ni_1_-N-C | Zn-CO_2_ | CO | __ | 93.4 | ^[10]^ |
| Ni_9_Cu_1_@NCNTs/CFM | Zn-CO_2_ | CO | 0.65 | 97 | ^[11]^ |
| MoS_2_/GF | Zn-NO | NH_3_ | 1.04 | 76.6 | ^[12]^ |
| Mo_2_C/Ni@C/CS | Zn-N_2_H_4_ | H_2_ | __ | 96 | ^[13]^ |
| Mo_2_C/NiCu@C | Zn-NH_3_ | H_2_ | __ | 91.6 | ^[14]^ |
| Ni-Doped-MoS_2_ | Zn-H_2_O | H_2_ | 3.3 | __ | ^[15]^ |
| Ni/Mo_2_C@CMF | Zn-Urea | H_2_ | 3.4 | 99 | **This work** |
| Ni/Mo_2_C@CMF | Zn-Urine | H_2_ | 2.8 | 98 |  |

**References:**

[1] G. Kresse and J. Furthmuller, *Comput. Mater. Sci*, 1996, **6**, 15-50.

[2] G. Kresse and J. Furthmuller, *Phys. Rev. B*, 1996, **54**, 11169-11186.

[3] J. P. Perdew, K. Burke and M. Ernzerhof, *Phys. Rev. Lett.*, 1996, **77**, 3865-3868.

[4] G. Kresse and D. Joubert, *Phys. Rev. B*, 1999, **59**, 1758-1775.

[5] P. E. Blochl, *Phys. Rev. B*, 1994, **50**, 17953-17979.

[6] J. F. Xie, X. Y. Wang, J. Q. Lv, Y. Y. Huang, M. X. Wu, Y. B. Wang and J. N. Yao, *Angew. Chem., Int. Ed.*, 2018, **57**, 16996-17001.

[7] X. Y. Wang, J. F. Xie, M. A. Ghausi, J. Q. Lv, Y. Y. Huang, M. X. Wu, Y. B. Wang and J. N. Yao, *Adv. Mater.*, 2019, **31**, 1807807.

[8] J. C. Hao, Z. C. Zhuang, J. C. Hao, K. C. Cao, Y. X. Hu, W. B. Wu, S. L. Lu, C. Wang, N. Zhang, D. S. Wang, M. L. Du and H. Zhu, *ACS Nano*, 2022, **16**, 3251-3263.

[9] F. Y. Wang, G. Wang, P. L. Deng, Y. Chen, J. Li, D. X. Wu, Z. T. Wang, C. T. Wang, Y. J. Hua and X. L. Tian, *Small*, 2023, **19**, 2301128.

[10] L. Jiao, J. T. Zhu, Y. Zhang, W. J. Yang, S. Y. Zhou, A. W. Li, C. F. Xie, X. S. Zheng, W. Zhou, S. H. Yu and H. L. Jiang, *J. Am. Chem. Soc.*, 2021, **143**, 19417-19424.

[11] S. J. Shen, C. Han, B. Wang and Y. D. Wang, *Chin. Chem. Lett.*, 2022, **33**, 3721-3725.

[12] L. C. Zhang, J. Liang, Y. Y. Wang, T. Mou, Y. T. Lin, L. C. Yue, T. S. Li, Q. Liu, Y. L. Luo, N. Li, B. Tang, Y. Liu, S. Y. Gao, A. A. Alshehri, X. D. Guo, D. W. Ma and X. P. Sun, *Angew. Chem., Int. Ed.*, 2021, **60**, 25263-25268.

[13] Y. Feng, Q. Shi, J. Lin, E. Chai, X. Zhang, Z. Liu, L. Jiao and Y. Wang, *Adv. Mater.*, 2022, **34**, 2207747.

[14] Y. Feng, L. Huang, Z. Xiao, X. Zhuang, T. S. Aslam, X. Zhang, Y. Tan and Y. Wang, *J. Am. Chem. Soc.*, 2024, **146**, 7771-7778.

[15] H. Wei, J. C. Si, L. B. Zeng, S. L. Lyu, Z. G. Zhang, Y. G. Suo and Y. Hou, *Chin. Chem. Lett.*, 2023, **34**, 107144.
